# Timing of phase‐amplitude coupling is essential for neuronal and functional maturation of audiovisual integration in adolescents

**DOI:** 10.1002/brb3.1635

**Published:** 2020-04-27

**Authors:** Takefumi Ohki, Takeru Matsuda, Atsuko Gunji, Yuichi Takei, Ryusuke Sakuma, Yuu Kaneko, Masumi Inagaki, Takashi Hanakawa, Kazuhiro Ueda, Masato Fukuda, Kazuo Hiraki

**Affiliations:** ^1^ Graduate School of Arts and Sciences University of Tokyo Tokyo Japan; ^2^ Department of Developmental Disorders National Institute of Mental Health National Centre of Neurology and Psychiatry Tokyo Japan; ^3^ Department of Neurosurgery Osaka University Graduate School of Medicine Suita Japan; ^4^ Osaka University Institute for Advanced Co‐Creation Studies Suita Japan; ^5^ Department of Mathematical Informatics Graduate School of Information Science and Technology University of Tokyo Tokyo Japan; ^6^ Mathematical Informatics Collaboration Unit RIKEN Center for Brain Science Saitama Japan; ^7^ College of Education Yokohama National University Yokohama Japan; ^8^ Department of Advanced Neuroimaging Integrative Brain Imaging Center National Centre of Neurology and Psychiatry Tokyo Japan; ^9^ Department of Psychiatry and Neuroscience Gunma University Graduate School of Medicine Gunma Japan; ^10^ Clinical Center for Developmental Disorders Shirayuri College Tokyo Japan; ^11^ Department of Neurosurgery National Center Hospital National Centre of Neurology and Psychiatry Tokyo Japan

**Keywords:** adolescence, audiovisual integration, delta‐beta coupling, neuronal oscillation, speech

## Abstract

**Objective:**

The ability to integrate audiovisual information matures late in adolescents, but its neuronal mechanism is still unknown. Recent studies showed that phase‐amplitude coupling (PAC) of neuronal oscillations, which is defined as the modulation of high‐frequency amplitude by low‐frequency phase, is associated with audiovisual integration in adults. Thus, we investigated how PAC develops in adolescents and whether it is related to the functional maturation of audiovisual integration. In particular, we focused on the timing of PAC (or the coupling phase), which is defined as the low‐frequency phase with maximum high‐frequency amplitude.

**Methods:**

Using magnetoencephalography (MEG) on 15 adults and 14 adolescents while they performed an audiovisual speech integration task, we examined PAC in association cortexes with a trial‐by‐trial analysis.

**Results:**

Whereas delta‐beta coupling was consistently observed in both adults and adolescents, we found that the timing of delta‐beta PAC was delayed by 20–40 milliseconds in adolescents compared with adults. In addition, a logistic regression analysis revealed that the task performance improves as the timing of delta‐beta PAC in the right temporal pole (TP) got closer to the trough position (180 degrees).

**Conclusion:**

These results suggest that the timing of PAC is essential for binding audiovisual information and underlies the developmental process in adolescents.

## Graphical abstract

Phase‐amplitude coupling (PAC) timing underlies maturation for audiovisual speech information integration. The timing of PAC was delayed 20–40 ms in adolescents compared to adults. The timing of PAC was a significant predictor of task performance.

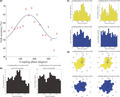

## 1 INTRODUCTION

Adolescence is a sensitive period in brain development. It has been well demonstrated that the adolescent brain shows dramatic changes in anatomical structures, such as white matter and gray matter (Gogtay et al., 2004; Paus, 2010), and also alterations in neurophysiological properties such as neural oscillations implemented with the balance of excitatory and inhibitory functions (Belelli et al., 2018; Brown, Herd, Belelli, & Lambert, 2015; Cho et al., 2015; Datta, Arion, & Lewis, 2015; Hashimoto et al., 2010; Vicini et al., 2001).

Neuronal oscillatory phenomena, such as cross‐frequency coupling (CFC) and intra‐frequency coupling (IFC), are key to elucidating the neuronal development in adolescents, as shown by a recent developmental cohort study (Cho et al., 2015). One of the most representative forms of CFC is the phase‐amplitude coupling (PAC), and it has been found to be a fundamental neuronal mechanism for encoding temporally sequential information, such as ordered locations (Dragoi & Buzsáki, 2006; Harris, Csicsvari, Hirase, Dragoi, & Buzsáki, 2003), and event sequences consisting of odor and sound stimuli that are unrelated to navigational information (Heusser, Poeppel, Ezzyat, & Davachi, 2016; Terada, Sakurai, Nakahara, & Fujisawa, 2017). On the other hand, IFC between brain regions is another important neuronal mechanism related to cognitive and developmental neurophysiological properties of adolescence (Doesburg, Tingling, MacDonald, & Pang, 2016; Uhlhaas et al., 2009). It is associated with functional brain networks, and its task‐dependent increase can aid the establishment of cognitive functions (Fries, 2005; Ohki et al., 2016). Of note, a recent study reported that CFC and IFC can coexist and their integration captures the multiplicity of human brain functions (Dimitriadis, 2018).

Existing studies on the development of PAC in humans mainly focus on the strength of PAC (scalar data) and few studies have investigated the timing of PAC (directional data), which is defined as the low‐frequency phase with a maximal high‐frequency amplitude. However, the timing of PAC provides unique information on brain function, which cannot be obtained by just focusing on the strength of PAC. For instance, a previous study demonstrated that each cell in the hippocampus and the entorhinal cortex has its own theta‐gamma coupling phase (Mizuseki, Sirota, Pastalkova, & Buzsáki, 2009; Valero et al., 2015, 2017; Valero & de la Prida, 2018): 180 degrees in the CA1, 90 degrees in the CA3 and dentate gyrus (DG), and 0 degrees in the entorhinal cortex layer 3. In addition, the coupling phase has been found to be associated with cognitive function (Latchoumane, Ngo, Born, & Shin, 2017) and aging (Helfrich, Mander, Jagust, Knight, & Walker, 2018).

Previous studies have demonstrated that multisensory integration, such as audiovisual integration, is important in the understanding of the neuronal mechanism of sensory perception (Ghazanfara & Schroederb, 2006), and revealed that PAC and the inter‐areal coordination between association cortexes, such as the temporal pole (TP) and posterior parietal cortexes, are associated with integration of audiovisual information in adults (Ohki et al., 2016; Ohki & Takei, 2018). Behaviorally, adolescents have a significantly lower performance level in audiovisual integration than adults (Ross et al., 2011), but the neuronal mechanism that explains this behavioral difference still remains elusive.

In this study, we investigated the neuronal and functional maturation of audiovisual integration in adolescence by focusing on the timing of PAC and IFC in association cortexes. We used magnetoencephalography (MEG) data from adults and adolescents obtained while they performed an audiovisual integration task. To appropriately analyze the PAC timing, we employed statistical methods tailored for directional data (Mardia & Jupp, 2008). As a result, we found that the timing of delta‐beta PAC in the right TP is essential for the binding of audiovisual information and underlies the developmental process in adolescents.

## 2 MATERIALS AND METHODS

### 2.1 Participants

Fourteen typically developing teenagers in late adolescence (mean age, 16.8 years; age range, 16–17 years; all boys, and all right‐handed; no teen girls consented to take part in our study) participated in the current study. Fifteen healthy adults (10 men and 5 women; mean age, 25.6 years; age range, 22–35 years; and all right‐handed) were enrolled in the control group. All participants had normal hearing and vision and no history of neurological, psychiatric, or developmental disorders. In this study, the nonrandom experimental design was adopted.

To define typical development, we used the Wechsler Adult Intelligence Scale III (WAIS‐III); the Social Communication Questionnaire (SCQ), Lifetime Version; and the Attention‐Deficit/Hyperactivity (ADHD) Rating Scale. All adolescents were characterized as developing typically if (a) they scored above 85 points in each of the subordinate four indexes of the WAIS‐III and (b) they met the standard criteria scores for the SCQ and the ADHD Rating Scale. All of the adolescents’ scores were within the normal ranges. Thus, none of the adolescents were excluded from our study.

To confirm that all the adult participants were healthy, we used the WAIS‐III, the Autism Questionnaire, and the ADHD Rating Scale. No adults were excluded from participation, and the data from all of the 15 adults were included in our analysis. Table S1 presents the characteristics of our participants (Appendix S1).

All participants provided informed written consent, and the experimental protocol (A2013‐051) was approved by the Ethics Committee of the National Institute of Mental Health, National Centre of Neurology and Psychiatry. Informed consent and the experimental protocols adhered to the tenets of the latest version of the Declaration of Helsinki.

### 2.2 Experimental design

Previous studies have demonstrated that watching mouth movements enhances speech comprehension, even in noisy environments. This phenomenon is the result of audiovisual integration, which is termed “the cocktail party effect” (Ohki et al., 2016; Ross et al., 2011; Golumbic et al., 2013). In the current study, we created original stimuli consisting of 100 movie clips of a professional female announcer that focused on mouth movements and the related speech. Each movie consisted of an emotionally neutral, six‐word sentence (2,460–2,780 ms long), such as the Japanese equivalent of “Megumi bought a yellow hat yesterday.” The words used in this experiment consisted of three to four morae to control the duration of the speech.

For each participant, the task included two movies that were presented simultaneously and contained different sentences that utilized the same syntactic structure (i.e., subject, transitive verb, adjective, noun, and adverb). We used Adobe Premiere Pro CS6 (Adobe, Inc.) to position the face in the center of the frame, equalize the relative size of the mouth, and clip the movies precisely. Audio signal levels (i.e., volumes) were measured as root‐mean‐square contrasts and were normalized using MATLAB R2017b (MATHWORKS, Inc.). The auditory signals were presented through in‐ear earphones (ER3‐A; Etymotic), so that the speech sounds were presented at a comfortable conversational level (i.e., a sound pressure level of 72 dB), and the visual stimuli were projected through an opening in the wall onto a back‐projection screen situated in front of the participants, who were inside a windowless shielded room. Before starting the experiment, the participants were required to fix their gaze on a fixation point on the screen and were asked whether they could clearly see the mouth movements in the movies using their peripheral vision. Each stimulus was located at a visual horizontal angle of −11° and a vertical angle of 10°. The participants were instructed to maintain their gaze on the fixation point during the video playback. All stimuli and triggers were controlled using Superlab 5 (Cedrus).

### 2.3 Experimental procedure

At the beginning of each trial (i.e., the preparatory period), instructions appeared in the center of the screen for 500 ms and indicated whether the participant should look to the left or right, and then the fixation point appeared for 500 ms (Figure S1). The two movies were then presented simultaneously. In Figure S1, the movie on the left‐hand side was the target, whereas the movie on the right‐hand side represented a proximate noisy environment (i.e., a distractor). After watching the instructions and the movies, two words were presented on the screen. The participants were told to indicate if the two words were presented in the sentence on the screen that they were instructed to direct their attention to by pressing a button. For example, for the sentence “Hiroto bought a yellow hat yesterday”, the two words may have been “Hiroto yesterday” (match/yes) or “Megumi yesterday” (mismatch/no). The sentences were not repeated across the trials.

It should be noted that focusing on the mouth movements was likely to be the only solution for comprehension of the target speech, because both movies showed utterances by the same woman. Additionally, the two auditory streams corresponding to the two movies were presented simultaneously, without lateralization, through both earphones. Therefore, before the experiment started, the participants were instructed to focus on the mouth movements to accomplish our goal of evaluating the ability of sequential audiovisual speech integration in late adolescence.

### 2.4 Magnetoencephalography and magnetic resonance imaging data collection

The MEG data were recorded in a magnetically shielded room with the high‐density whole‐scalp VectorView MEG System (Elekta‐Neuromag), which contains a magnetometer and two orthogonal planar gradiometers at each of the 102 positions (306 sensors in total). We recorded MEG data for each participant during the task. Data were sampled at 1,000 Hz with a band‐pass filter from 0.03 Hz to 330 Hz. We recorded the positions of four head position indicator coils, the nasion, the right and left preauricular points, and more than 100 additional points randomly dispersed over the scalp using a 3‐dimensional (3D) digitizer (Fastrak Polhemus). The head position was continuously monitored with the head position indicator coils, which allowed for movement compensation throughout the recording session.

It has been reported that saccadic eye movements cause noise, especially in the high‐frequency bands (Gawne, Killen, Tracy, & Lahti, 2017). Therefore, it was necessary to control for saccadic eye movements during our tasks and analyses. Both electrocardiography (ECG) and electrooculogram (EOG) signals were recorded to detect trials containing heartbeats, vertical and horizontal eye movements, and blink artifacts. Also, 10 min of data from a vacant room were recorded and used for noise normalization. Structural T1‐weighted magnetic resonance imaging (MRI) scans were obtained for all participants using a 3T MRI scanner (3T Verio; Siemens) with a 12‐channel phased‐array receiver coil, and a 1 mm × 1 mm × 1 mm sized voxel was acquired using a 3D magnetization‐prepared rapid gradient echo sequence (repetition time, 1,900 ms; echo time, 2.52 ms; flip angle, 9°; acceleration factor 3*D* = 1).

### 2.5 Data preprocessing

To remove external noise and correct for head movements, the temporal extension of signal‐space separation (Taulu & Simola, 2006), which is implemented in MaxFilter 2.0 (Elekta‐Neuromag), and notch filtering were applied to the data to remove alternating currents of 50, 100, 150, 200, and 250 Hz. Two uncontrollable channels identified via visual inspection were rejected as bad channels. All the data were analyzed using MATLAB R2017b (MathWorks), Brainstorm (Tadel, Baillet, Mosher, Pantazis, & Leahy, 2011), and FreeSurfer (Athinoula A. Martinos Center for Biomedical Imaging; Destrieux, Fischl, Dale, & Halgren, 2010). As presented in Figure S1B, the task‐related MEG data were split into trials lasting 3,900 ms that encompassed the “inter‐trial interval” (500 ms), the “attentional direction” cues (500 ms), the “fixation point” (500 ms), and the “movie” (2,400 ms).

Seventy trials were collected for each participant. Among the trials, we defined the “movie” as the analyzed time course (0–2400 ms) for our analysis. Trials were rejected if the peak exceeded 50 μV, 80 μV, 1,000 fT, or 3,000 fT/cm in the ECG, EOG, magnetometer, or the gradiometer channels, respectively. Furthermore, the data segment above the threshold of two standard deviations (*SD*s) for EOG and four *SD*s for ECG measurements during the trials was detected with signal‐space projections, confirmed via visual inspection, and removed. These manipulations resulted in the exclusion of 1–8 trials for each condition per participant.

To address the data size fairly and maintain a constant signal‐to‐noise ratio across all the participants and conditions, we selected and analyzed 60 trials per participant. For participants who had more than 60 unrejected trials, we randomly chose 60 of these trials. The same set of trials was used for all analyses for each participant. For each participant, FreeSurfer (Athinoula A. Martinos Center for Biomedical Imaging) was used to create dense triangulations of cortical surface data (Destrieux et al., 2010) that were based on T1‐weighted image data. These data were co‐registered with the standard brain (MNI305 [FsAverage]; Montreal Neurological Institute) using a spherical representation of the cortex (Fischl, Sereno, Tootell, & Dale, 1999). Brainstorm (Tadel et al., 2011) was then used to downsample the number of vertices from 287,802 to 15,002. This corresponded to a spacing of approximately 0.3 cm^2^ per vertex.

### 2.6 Source estimation

Source reconstruction was conducted using the minimum‐norm estimate (MNE) method. The MNE method makes minimal assumptions about the generators of brain activity (Hämäläinen & Ilmoniemi, 1994), and provides an optimal source estimation for analyzing complicated or totally unknown sources within the general spatial resolution limits of MEG measurements. By using the overlapping spheres model, an inverse solution was obtained based on the forward solution of the lead field matrix, which models the signal pattern generated by a set of electric dipoles, on the surface of the cortex. This model is based on the estimation of a different sphere for each sensor and can estimate a magnetic field that locally fits the shape of the head in the surroundings of each sensor. To compute noise normalization, a noise covariance matrix was created using empty room data that were recorded for 10 min before the experiment. The MNE was applied to each individual data set, and individual source activation estimations were transformed to the standard brain.

### 2.7 Regions of interest

As regions of interest (ROIs), we focused on seven regions in the association cortex related to audiovisual integration (Driver & Noesselt, 2008; Stein & Stanford, 2008). Specifically, we analyzed 594 vertices of the TP (left, 278 vertices; right, 316 vertices), 169 vertices of the superior temporal sulcus (STS; left, 96 vertices; right, 73 vertices), 410 vertices of the caudal middle frontal region (CMF; left, 196 vertices; right, 214 vertices), 884 vertices of the inferior parietal region (IP; left, 428 vertices; right, 456 vertices), 874 vertices of the superior parietal region (SP; left, 439 vertices; right, 435 vertices), 628 vertices of the supramarginal region (SM; left, 301 vertices; right, 327 vertices), and 289 vertices of the pars opercularis region (PO; left, 153 vertices; right, 136 vertices). These regions are illustrated in Figure S2. In the following analysis, we considered all 14 regions as ROI.

### 2.8 Phase‐amplitude coupling evaluation

**FIGURE 1:**
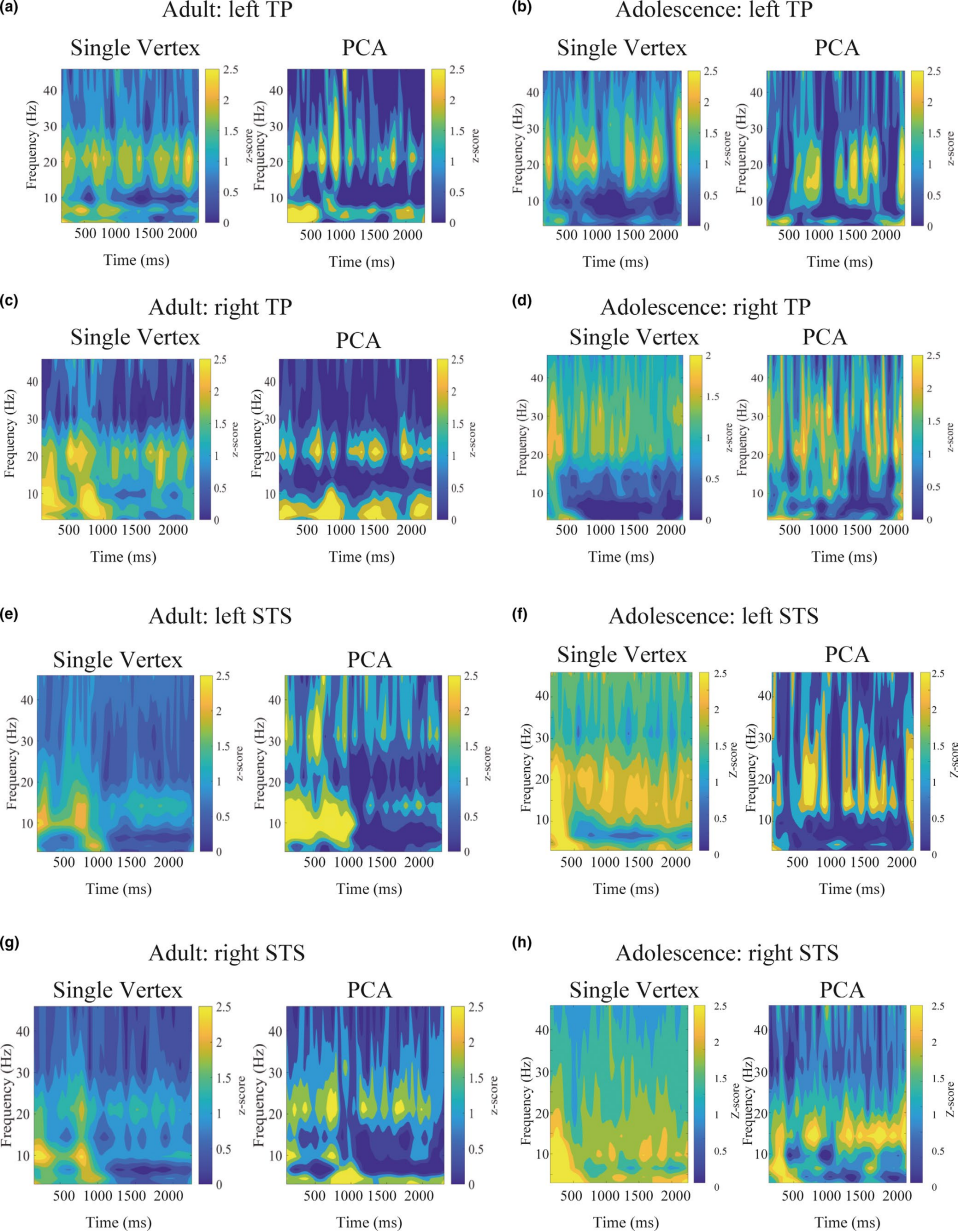
The time‐frequency power of a single vertex and a principal component (PC) of the temporal pole (TP) and the superior temporal sulcus (STS). This calculation was conducted with the complex Morlet wavelet convolution. (a) Adult single vertex and PC of an adult left TP, (b) Adolescent single vertex and PC of the left TP, (c) Adult single vertex and PC of an adult right TP, (d) Adolescent single vertex and PC of the right TP, (e) Adult single vertex and PC of an adult left STS, (f) Adolescent single vertex and PC of the left STS, (g) Adult single vertex and PC of the right STS, and (h) Adolescent single vertex and PC of the right STS. The time‐frequency power at the single vertex level is depicted based on the average data of all the participants in each group. The time‐frequency power of one representative PC in a single subject is shown. Note that a similar result is obtained even when using different space data, that is, single vertex or region of interest (ROI) base calculation. In these figures, the *x*‐ and *y*‐axis denote time (ms) and frequency (Hz), respectively. Each frequency power was normalized (*z*‐score) using the baseline. These results strongly suggest that neuronal activation patterns in TP and STS are associated with the power fluctuation of beta and gamma. Note that the figures of the PC were created based on one single subject, since the PC cannot be averaged across all participants

In order to quantify the change in amplitude for each frequency band, we applied the complex Morlet wavelet convolution (Cohen, 2014) to each vertex. In this calculation, we divided frequencies from 3 to 50 Hz into eight logarithmically spaced bands. The outputs of the complex Morlet wavelet convolution were standardized for each vertex, where the mean and standard deviation in the period of 500 ms before stimulus onset was used as a reference. For each vertex, the *z*‐score was averaged over all the participants. Thus, a representative power time series was obtained for each vertex. To confirm the above calculations (the single vertex base calculation) successfully extracted some representative neuronal features based on a broader cortical space, we also conducted the principal component analysis (PCA) to extract the principal component (PC) waveform as a representative feature of each ROI. The selection of the number of PCs was determined by the 95% cumulative contribution rate (%) for the data, that is, we used five PCs. For these five PCs in each ROI, we conducted the complex Morlet wavelet convolution to obtain the frequency power fluctuation in the time series (Figure 1, Figure [Link], [Link], [Link]).

**FIGURE 2:**
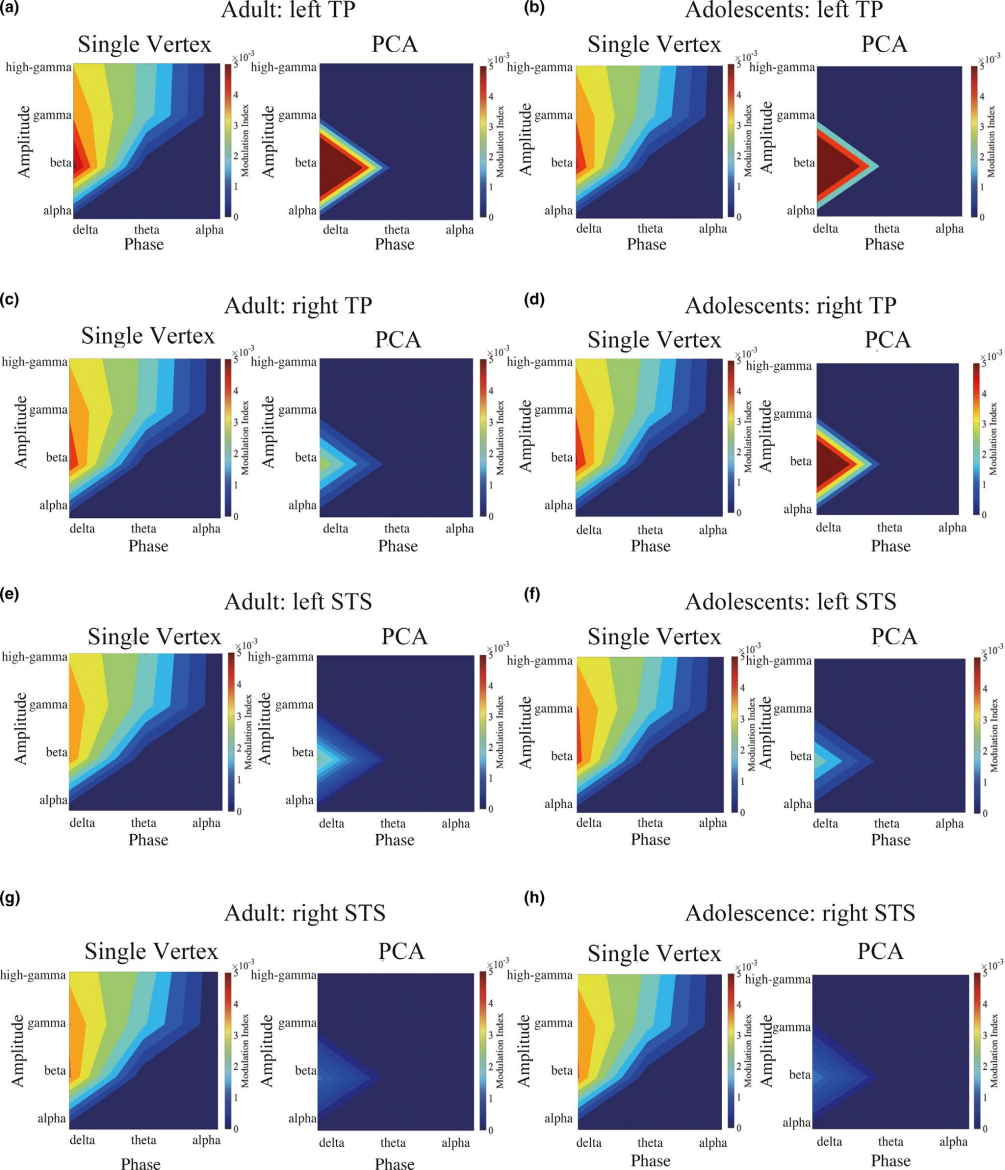
Comodulogram of the modulation index (MI) in the temporal pole (TP) and superior temporal sulcus (STS). In this figure, we plotted the MI for the bilateral TP and STS. (a) Adult single vertex and principal component (PC) of the left TP, (b) Adolescent single vertex and PC of the left TP, (c) Adult single vertex and PC of the right TP, (d) Adolescent single vertex and PC of the right TP, (e) Adult single vertex and PC of an adult left STS, (f) Adolescent single vertex and PC of the left STS, (g) Adult single vertex and PC of the right STS, and (h) Adolescent single vertex and PC of the right STS. These figures show that the strongest coupling pattern was delta‐beta coupling in the TP and STS in both groups. We calculated MI in two ways; namely at the single vertex base in order to elucidate more detailed spatial information and the PC as a more general representation of the spatially distributed information in TP and STS. For demonstration purposes, we arbitrarily selected one typical activation pattern from one vertex and one component in each region of interest (ROI). Note that in this calculation delta and theta bands were used for the phase data. For the amplitude data, beta, gamma and high‐gamma were utilized

Second, we evaluated the statistical significance of PAC in each vertex, each trial, and each participant for the following five frequency band combinations of typical coupling patterns: (1) delta‐beta coupling (3–5 Hz/12–30 Hz), (2) delta‐gamma coupling (3–5 Hz/30–50 Hz), (3) delta‐high gamma coupling (3–5 Hz/50–100 Hz), (4) theta‐gamma coupling (5–8 Hz/30–50 Hz), and (5) theta‐high gamma coupling (5–8 Hz/50–100 Hz). The PAC strength in each vertex, trial, and participant was quantified using the modulation index (MI) introduced by Tort, Komorowski, Eichenbaum, and Kopell (2010) with 18 phase bins that consisted of 20 degrees. In addition to calculating the MI value for the real data, we randomly shuffled the phase time series 200 times and calculated 200 surrogate MI values. If the realized MI value was larger than the maximum surrogate MI value, then the PAC was considered statistically significant at *p* < .005. Please note that we obtained a single MI value per trial (the MI value is not time series data). These MI values were used to determine which combinations of frequency bands formed PAC in our data (Figure 2, Figure [Link], [Link], [Link]) and what percentage of all trials was statistically significant (Table S2). We did not correct for multiplicity of testing, which requires a large amount of surrogate data, due to limited computational resources. To calculate MI, we used two types of data, that is, the source waveform at the single vertex level and the PC time series data.

### 2.9 Phase‐amplitude coupling timing

For each vertex, trial, participant, and frequency band combination, we calculated the PAC timing (coupling phase). The PAC timing is defined as the low‐frequency phase (delta and theta) at which the high‐frequency amplitude (beta, gamma, and high‐gamma) is at its maximum. We hypothesized that the most suitable coupling phase to accomplish cognitive function would be common across the participants. Thus, we explored vertices where the coupling phase was consistent in each group (i.e., the adult group and adolescent group) using the Rayleigh test (Mardia & Jupp, 2008). For each of the seven brain regions, we identified vertices with *p* < .05 (Bonferroni correction; the p‐values were multiplied by the number of vertices) and marked them as ROIs. Additionally, to determine whether the consistency of the coupling phase occurred in the task‐dependent manner, we conducted a Rayleigh test for 2 min when the participants’ eyes were open, producing resting state data for both groups in the bilateral TP.

To compare the distribution of the coupling phase over the ROI between adults and adolescents, we calculated the mode and the circular variance of the coupling phase in each region. We also applied the likelihood‐ratio test for multinomial distributions to determine whether the distributions of the coupling phase were significantly different between adults and adolescents.

### 2.10 Logistic regression analyses with the coupling phase and phase‐locking value

We conducted logistic regression analyses to examine whether the coupling phase was significantly associated with the ability to integrate audiovisual information. The participants attempted the audiovisual integration task 60 times, and each trial resulted in success or failure. Thus, we modeled the success probability (*p*) of each trial using the following equation:

$$\log\frac{p}{1-p}=\beta_{0}+\beta_{1}\cos\left(\theta -\theta_{0}\right)$$

where θ is the coupling phase of the trial and $\left(\beta_{0},\beta_{1},\theta_{0}\right)$ are parameters that are determined from the data. The parameter $\beta_{0}$ is the intercept term, and a value of $\beta_{1}>0$ determines the degree that the ability to integrate sequential audiovisual information depends on the coupling phase. The parameter $\theta_{0}$ determines the coupling phase with the maximum success probability. Therefore, this formula signifies that the success probability is larger for trials with a coupling phase closer to the optimal coupling phase (i.e., $\theta_{0}$ ). For parameter estimation, we fitted $\left(\beta_{0},\beta_{1}\right)$ with the MATLAB function, *glmfit*, while selecting $\theta_{0}$ from 18 candidates corresponding to each 20‐degree segment to maximize the log‐likelihood.

To determine that the vertices that were not included in a ROI were unrelevant for the integration of audiovisual information, we conducted the aforementioned logistic regression analysis for all vertices (e.g., 278 vertices in the left TP). In addition, to determine whether the optimal coupling phase was common across all the adults and adolescents, we conducted the aforementioned analysis in two manners: (1) assuming common parameters $\left(\beta_{0},\beta_{1},\theta_{0}\right)$ for the adults and adolescents and (2) assuming separate parameters $\left(\beta_{0},\beta_{1},\theta_{0}\right)$ for the adults and adolescents respectively.

We also conducted a logistic regression analysis to investigate whether the phase synchronization between ROIs was significantly associated with the ability to integrate audiovisual information. To quantify the phase synchronization, we used the phase‐locking value (PLV), which is calculated by the following four steps. First, we calculated the representative waveforms using the PCA. The selection of the number of PCs was determined by the cumulative contribution rate (%). Namely, we chose five PCs that reached a cumulative contribution rate of 95%. Second, we extracted time‐frequency data from delta to high‐gamma on each component, using eegfilt (Delorme & Makeig, 2004). Third, we calculated the time series of phase, using Hilbert transform. Fourth, PLV was performed. Please note that PLV is utilized as a measurement of the inter‐site synchrony here; namely, this calculation was conducted pairwise between regions. Here, we set the seed region to the right TP based on the results of the logistic regression analysis with coupling phase, that is, the networks between the right TP and the other 13 areas (all ROIs) were calculated. Then, by using the PLV as a covariate, we conducted logistic regression analyses to explore regions for which the phase synchronization with the right TP was significantly associated with the ability to integrate audiovisual information.

## 3 RESULTS

### 3.1 Phase‐amplitude coupling evaluation

Morlet wavelet analysis revealed a strong amplitude modulation of high‐frequency bands such as beta and gamma (Figure 1, Figure [Link], [Link], [Link]). These activations were not transient but sustained during the audiovisual integration task. Also, these amplitude fluctuations were not region‐specific, but rather similarly observed in all ROIs.

Based on the strong amplitude modulation of beta and gamma, we examined the significance of PAC using the method established by Tort et al. (2010). We found that the PAC was significant for all five frequency band combinations (i.e., delta‐beta, delta‐gamma, delta‐high gamma, theta‐gamma, and theta‐high gamma). Most trials (see Table 1 and Table S2) showed significant MI values (*p* < .005) for these five frequency band combinations in all 14 brain regions.

**TABLE 1:** Percentage of trials with a significant modulation index in the temporal pole and superior temporal sulcus

Adult		Adolescent
TP	Delta‐beta 99.28 (left) and 99.34 (right)	Delta‐beta 99.36 (left) and 99.35 (right)
	Theta‐gamma 98.75 (left) and 98.77 (right)	Theta‐gamma 98.77 (left) and 98.79 (right)
STS	Delta‐beta 99.38 (left) and 99.31 (right)	Delta‐beta 99.33 (left) and 99.34 (right)
	Theta‐gamma 98.66 (left) and 98.69 (right)	Theta‐gamma 98.79 (left) and 98.68 (right)

Abbreviations: STS, superior temporal sulcus; TP, temporal pole.

### 3.2 Phase‐amplitude coupling timing

**FIGURE 3:**
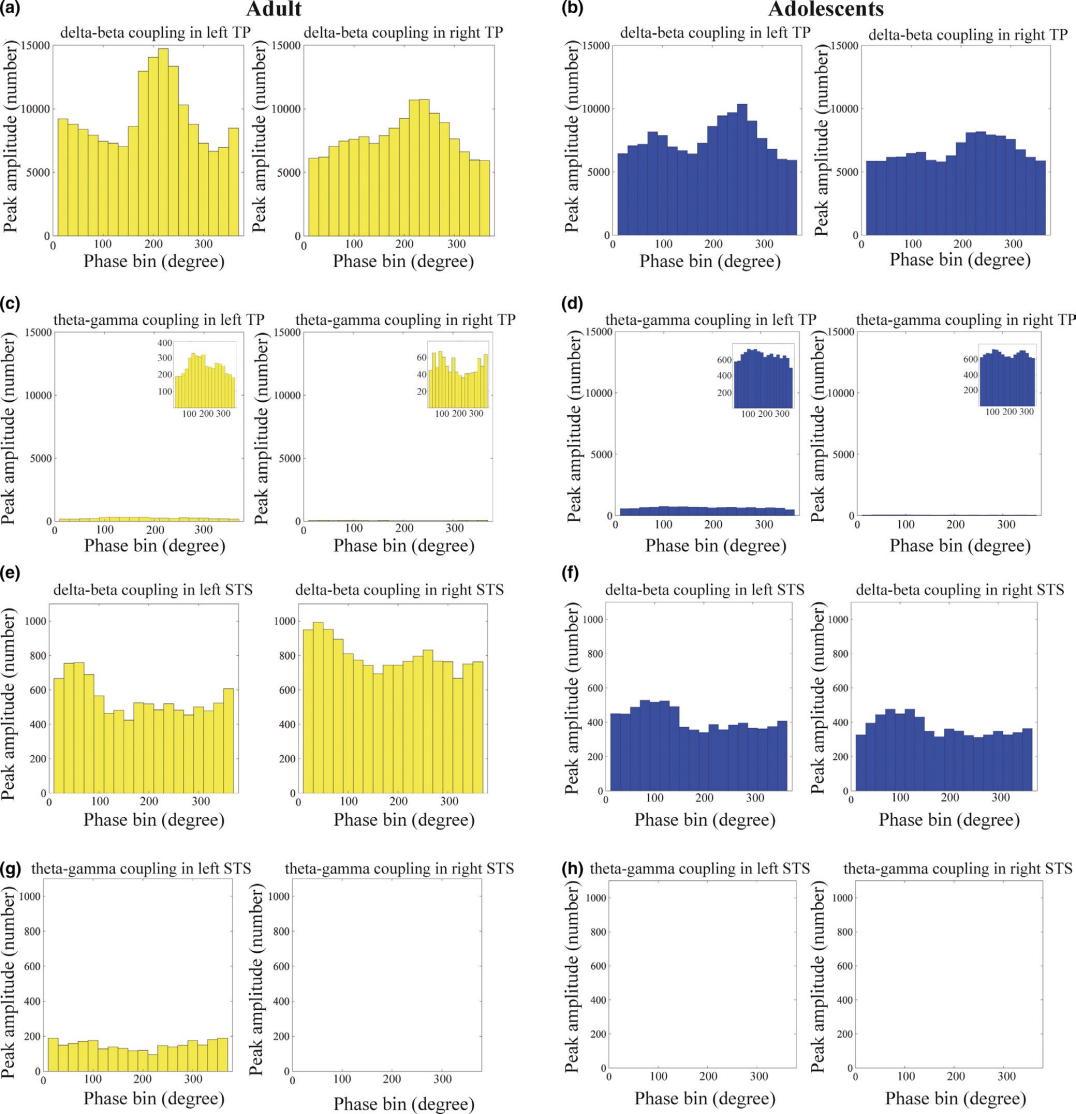
Histograms of the vertices showing delta‐beta coupling and theta‐gamma coupling in the temporal pole (TP) and the superior temporal sulcus (STS). The yellow and blue histograms present the findings for the adults and adolescents, respectively. (a) The left histogram represents the number of trials with beta amplitude modulation for the delta phase in the regions of interest (ROIs) in the left TP. The right histogram similarly represents the number of trials showing delta‐beta coupling in the ROIs in the right TP. (b) The blue histogram indicates the number of trials demonstrating delta‐beta coupling in the adolescent group. The left histogram presents the results of the ROIs in the left TP, and the right histogram presents the results of the ROIs in the right TP. (c and d) Theta‐gamma coupling in the ROIs in the bilateral TP is depicted for the adults and adolescents in the left and right histograms, respectively. Fewer vertices are detected in theta‐gamma coupling than in delta‐beta coupling. This tendency occurred similarly in both groups. (e and f) The number of trials showing delta‐beta coupling in the ROIs in the bilateral STS is depicted. Panel e presents the results of the adult group and panel f presents the findings of the adolescent group. The mode of the coupling phase in the left TP and right TP is approximately 220 degrees and 240 degrees, respectively, whereas it is 60 degrees and 40 degrees in the left STS and right STS, respectively. (g and h) The number of trials showing theta‐gamma coupling in the STS is presented. The traits are identical with those of the TP; very few or no trials with theta‐gamma coupling are detected in adults or adolescents. Note that these figures were depicted to show the peak of the coupling phase (the scale of the *y*‐axis is adjusted for illustration purposes)

Next, we calculated the coupling phase for each of the five frequency band combinations. For example, the coupling phase for delta‐beta coupling is defined as the delta phase with the maximum beta amplitude. We then applied the Rayleigh test to determine whether the coupling phase was consistent across all the trials and individuals in both groups. According to our results, the coupling phase was only consistent for the delta‐beta coupling. In particular, in the adult group, 187 vertices in the left TP, 147 in the right TP, 11 in the left STS, and 13 in the right STS were significant (*p* < .05, Bonferroni correction) for delta‐beta coupling (Figure 3a,e); however, very few vertices were significant in the TP (left, 5; right, 0) and the STS (left, 11; right, 16) for theta‐gamma coupling (Figure 3c,g). Similar to the adult group, the following vertices were significant (*p* < .05, Bonferroni correction) for delta‐beta coupling in the adolescent group: 164 vertices in the left TP, 144 in the right TP, nine in the left STS, and eight in the right STS (Figure 3b,f). However, we observed only a small number of vertices that had theta‐gamma coupling (Figure 3d,h). Similar results were obtained for other regions and other frequency band combinations (see Figure S5 and Table S3). Based on these results, we focused on the delta‐beta coupling in the following analysis. Please note that we set the y‐axis ranges for each ROI (e.g., for TP and for STS). For instance, the y‐axis was set at 15,000 for the TP region and 1,200 for the STS region for both coupling patterns, since we can assume that the number of peak positions (the *y*‐axis range) was maintained in the ROI and not in the coupling pattern. Therefore, we set the identical *y*‐axis range to detect the most consistent coupling phase across the trials in each ROI. However, this manipulation hinders detection of the peak position of theta‐gamma coupling for readers. Then, we added an inset into the figure in which we changed the y‐axis ranges for theta‐gamma coupling, allowing the peak position to be easily detected (Figure 3c,d, Figure S5A, B, D, H, and R). Additionally, to determine whether the consistency of the coupling phase occurred in a task‐dependent manner, we conducted a Rayleigh test for 2 min when the participants’ eyes were open, producing resting state data for both groups in the bilateral TP. However, this test only detected a single vertex in the left TP of the adults. Compared with the number of vertices in the task‐related data, we concluded that the consistency of the coupling phase mainly depended on the task‐related brain activities.

**FIGURE 4:**
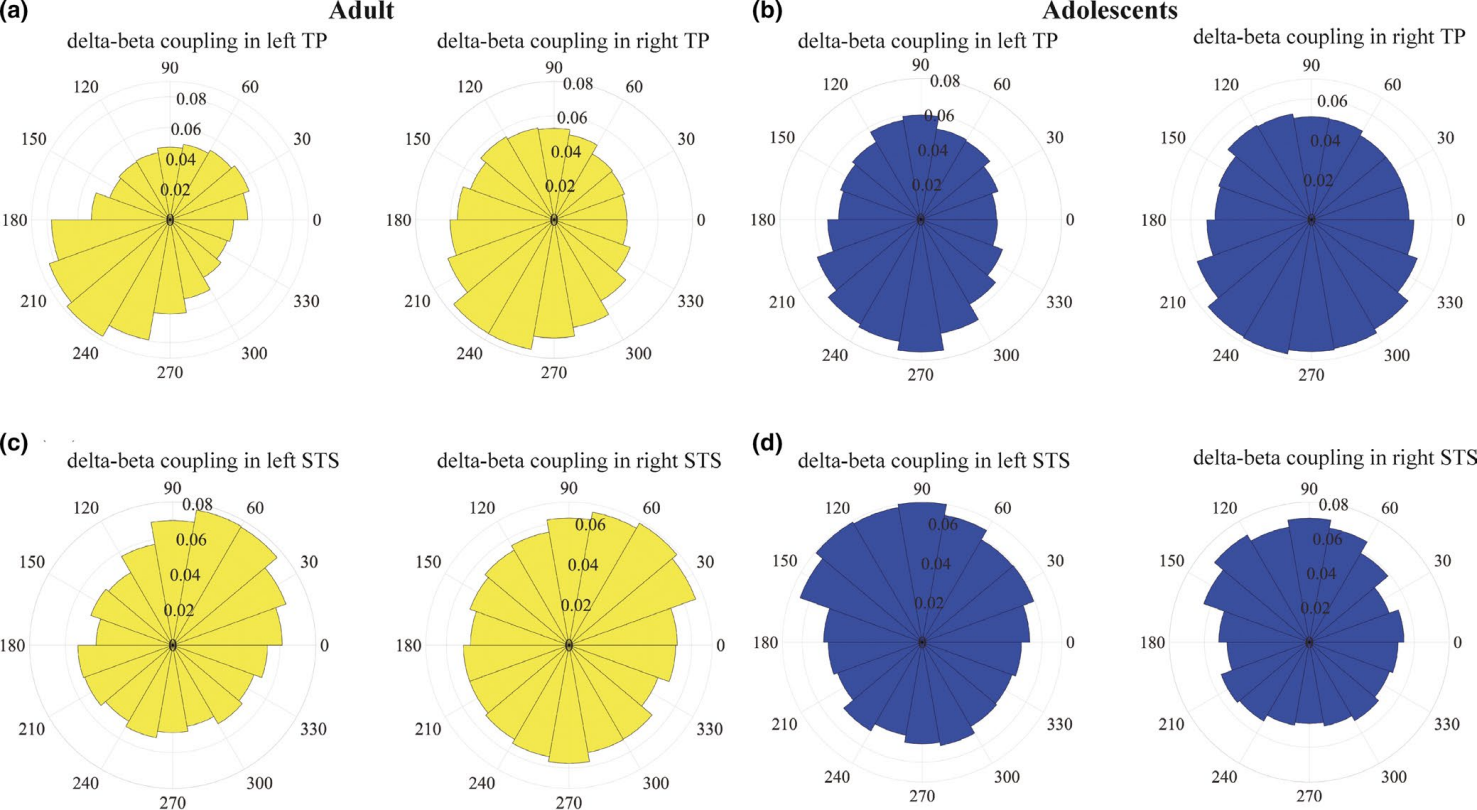
Rose plots of the delta‐beta coupling phase in the regions of interest (ROIs). The yellow and blue plots present the results of the adults and adolescents, respectively. (a) The delta‐beta coupling phase in the bilateral temporal pole (TP) is presented. The adult group (yellow plots) shows a clear peak in phase coupling at 220 degrees in the left TP and 240 degrees in the right TP. The inner number is the probability (i.e., the number of trials in each bin divided by the total number of trials in the TP). (b) The delta‐beta coupling phase in the bilateral TP in adolescents is presented. In the adolescent group, the mode of the coupling phase is approximately 270 degrees in the left TP and 240 degrees in the right TP. (c) The delta‐beta coupling phase in the superior temporal sulcus (STS) in the adults. The coupling phase in the STS is approximately 60 degrees, which occurs much earlier than the coupling phase in the TP. (d) The coupling phase in the STS in adolescents. Consistent with the results in adults, the coupling phases occur much earlier in the bilateral STS than the coupling phases in the TP. The difference in the coupling phase between these two groups, in particular, the coupling phase in STS in adolescents, is approximately 80 degrees, which suggests that the coupling phase in adolescents is delayed by 20–40 ms, compared with the coupling phase in adults

We calculated the mode and circular variance of the coupling phase for delta‐beta coupling in the TP and STS (Figure 4). The mode of the coupling phases in the left TP and right TP in the adult group was 220 degrees and 240 degrees, respectively, and the circular variance was 0.13 and 0.11, respectively (Figure 4a). However, the mode of the coupling phases in the left STS and right STS was 60 degrees and 40 degrees, respectively, and the circular variance was 0.097 and 0.047, respectively (Figure 4c). In the adolescent group, the mode of the coupling phase in the left TP and right TP was 260 degrees and 240 degrees, respectively, and the circular variance was 0.059 and 0.074, respectively (Figure 4b). The mode of the coupling phases in both the left STS and right STS was 80 degrees and the circular variance was 0.10 and 0.20 in the left STS and right STS, respectively (Figure 4d). Results from a likelihood‐ratio test for multinomial distributions revealed that the differences in distribution of the coupling phase between adults and adolescents were significant for all vertices in the ROI (*p* < 10^–6^). Therefore, the delta‐beta coupling phase was significantly different between adults and adolescents. Thus, the timing of the delta‐beta coupling in adolescents was delayed by approximately 20–40 ms compared with the adults (Figure 4 presents the delta‐beta coupling in the TP and the STS, Figure S6 presents the delta‐beta coupling in the remaining five brain regions).

### 3.3 Logistic regression analysis with the coupling phase and PLV

**FIGURE 5:**
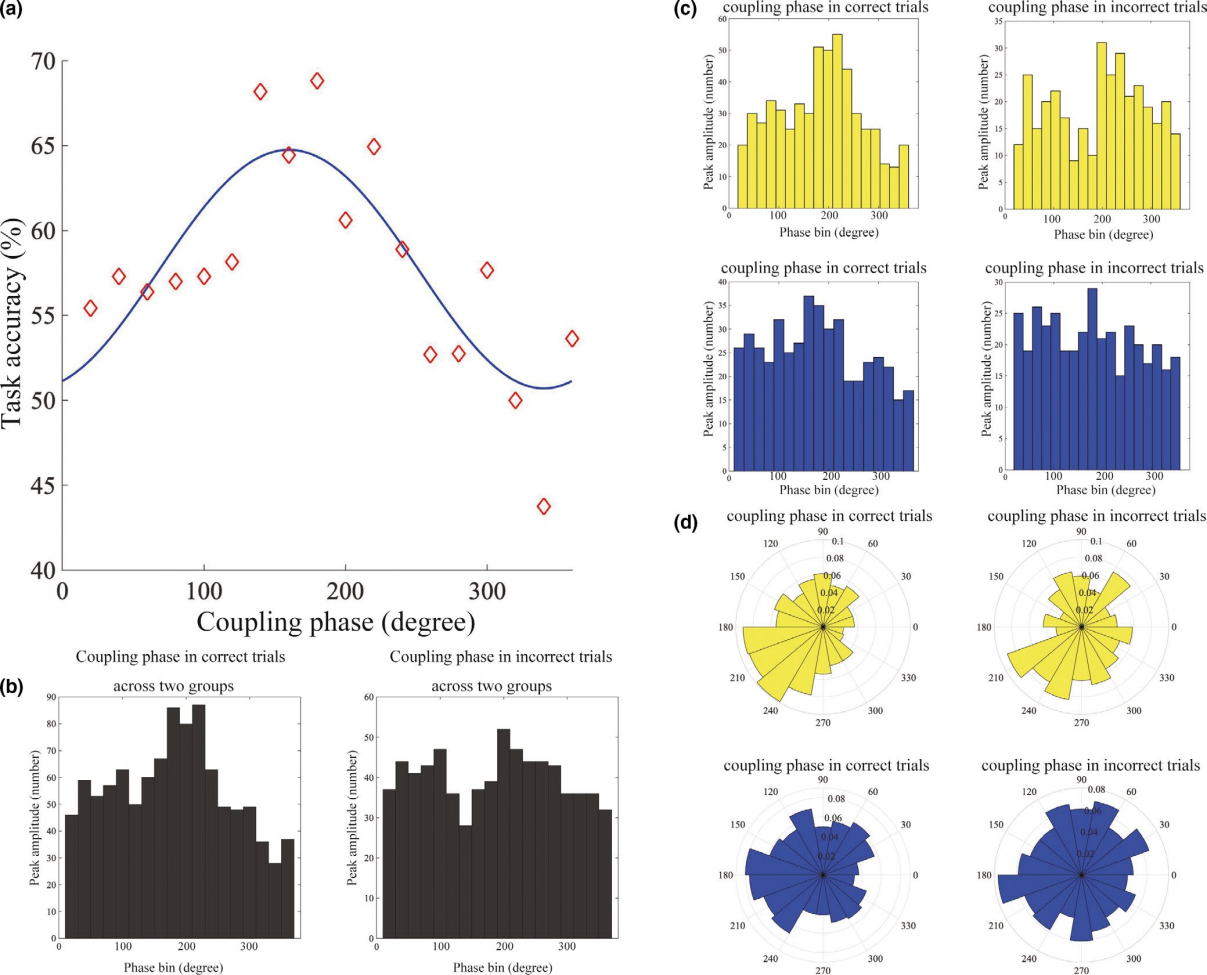
The findings of the logistic regression analysis, focusing on the relationship between the coupling phase and task performance. (a) The dark blue line indicates the expected task performance for each coupling phase, that was estimated using logistic regression. The maximum task accuracy appears at approximately 180–220 degrees, which is approximately the trough position of the delta phase. The red diamonds represent the task accuracy at each coupling phase across the two groups. (b) The black histograms present the coupling phase at the correct and incorrect trials across the two groups. The coupling phase at the correct trials is on the left, and the coupling phase at the incorrect trials is on the right. A more discernible peak is visible in the correct trials. (c) The histograms of the coupling phase at the correct trials (left) and the incorrect trials (right) are shown. The correct trials and incorrect trials in the single vertex that showed a significant relationship between the coupling phase and task performance are presented separately. The yellow and blue histograms denote the coupling phase in the adult and adolescent groups, respectively. (d) The rose plots of the coupling phase in the correct and incorrect trials. The upper yellow rose plots represent the coupling phase of the adults. In the correct trials, the peak occurs at 180–220 degrees; however, in the incorrect trials, the peak tends to be delayed by 20–40 degrees. In the adolescents, the coupling phase in the correct trials tends to gather at 180–240 degrees, which is an identical pattern with that of the adults. In contrast, the distribution of the coupling phase in the incorrect trials is scattered or does not have a clear peak for the delta phase. The inner numbers in panel d indicate the probability (i.e., the number of trials in each bin divided by the total number of trials in a single vertex)

We investigated whether the coupling phase was a significant factor for predicting the performance on audiovisual integration tasks using a logistic regression analysis (Figure 5). We explored vertices with a coupling phase that was significantly associated with the task performance. As a result, two vertices in the right TP were significant (*p* < .05, with Bonferroni correction). These vertices belonged to an ROI detected with the Rayleigh test, and the estimate of the optimal (i.e., best task performance) coupling phase was approximately 160–200 degrees. Thus, trials with the delta‐beta coupling phase close to the trough position had significantly higher scores than those with the delta‐beta coupling phase further away from the trough (Figure 5a). We confirmed that the optimal coupling phase identified by logistic regression was not affected within or across the groups. These findings suggested that delta‐beta coupling at the trough position in the right TP may be important for the integration of audiovisual information.

We investigated if the distribution of the coupling phase differed between correct trials and error trials in the significant vertex of the TP, indicated by the logistic regression analysis within and across the groups. Figure 5b separately presents the distributions of the delta‐beta coupling phases in the TP for the correct and incorrect trials. The coupling phases of the correct trials were concentrated at approximately 180–220 degrees, whereas the coupling phases of the incorrect trials were more widely scattered, and a clear peak was not observed. These results also suggest that the delta‐beta coupling at the trough position in the TP may be important for the integration of audiovisual information. Similar traits were also revealed in the plots depicting data for adults and adolescents separately (Figure 5c). Figure 5d presents the rose plots for the coupling phase in the correct and incorrect trials for adults and adolescents separately. These data also imply that PAC timing may be important for audiovisual integration tasks.

Finally, logistic regression analysis with PLV demonstrated that theta oscillation between the right TP (the seed regions) and the left CM, and the right SP was significantly correlated with task accuracy in the adults only and not in the adolescents (*p* < .05 with Bonferroni correction, Figures S7 and S8). These results suggest that PAC, as a representative of CFC and IFC, was coexisting and worked cooperatively (Dimitriadis, 2018; Ohki et al., 2016).

## 4 DISCUSSION

In this study, we investigated the functional maturation of audiovisual integration in adolescents by focusing on PAC timing in association cortexes. First, we found that the most prominent coupling pattern during the audiovisual integration task in both adolescents and adults was delta‐beta coupling, not theta‐gamma coupling. Next, we examined the delta‐beta PAC timing (or coupling phase) and found that delta‐beta PAC is delayed by 20–40 ms in adolescents compared with adults. Finally, we conducted a logistic regression analysis in a trial‐by‐trial manner, which demonstrated that the delta‐beta coupling phase was a significant predictor of the task performance, with the optimal coupling phase at approximately 180 degrees (trough position). To the best of our knowledge, this is the first study to reveal that PAC timing may be involved in neuronal and functional maturation of audiovisual integration in adolescents.

### 4.1 Diversity of the coupling phase and its physiological role

By examining association cortexes associated with multisensory processing (Driver & Noesselt, 2008; Ohki et al., 2016; Perrodin, Kayser, Abel, Logothetis, & Petkov, 2015; Perrodin et al., 2014, 2015; Straube, Wroblewski, Jansen, & He, 2018), we found that the coupling phase of delta‐beta PAC varies among brain regions. This finding is consistent with the theta‐gamma coupling phases in the hippocampus and the entorhinal cortex (Mizuseki et al., 2009): 180 degrees in the CA1, 90 degrees in the CA3 and DG, and 0 degrees in the entorhinal cortex layer 3. Moreover, recent studies have indicated that each cell type has its own unique coupling phase of the theta wave (Valero et al., 2015, 2017; Valero & de la Prida, 2018). For instance, at least two gamma‐aminobutyric acid (GABA)ergic interneuron types, such as parvalbumin (PV) and somatostatin (SOM), have a significant role in delta‐beta coupling (Chen et al., 2017; Ohki & Takei, 2018; Veit, Hakim, Jadi, Sejnowski, & Adesnik, 2017). Overall, these previous findings and our current results suggest that each brain region or subregion has its own unique coupling phase. Further investigation into the spatial distribution of the coupling phase may elucidate neuronal circuit properties and functionality implemented via PAC.

The coupling phase has been found to be relevant to memory function (Latchoumane et al., 2017) and aging (Helfrich et al., 2018). In this study, we demonstrated that the coupling phase in the TP is a significant predictor of task performance by a logistic regression analysis. The optimal coupling phase was approximately 180 degrees (trough position). Namely, the success probability of audiovisual integration tasks increases as the coupling phase gets closer to the trough position. Interestingly, the CA1 cell assemblies, which have the richest spatial information of the current position, also show phase‐locked firing at the trough of theta oscillations (Brown, Frank, Tang, Quirk, & Wilson, 1998; Dragoi & Buzsáki, 2006; Kloosterman, Layton, Chen, & Wilson, 2014; McHugh, Blum, Tsien, Tonegawa, & Wilson, 1996). In future studies it would be interesting to investigate the physiological significance of PAC at the trough position.

We also found that a low‐frequency phase, such as the theta‐phase, between the seed region (the right TP) and the left CM, and the right SP was positively correlated with task accuracy in the adult group only (Figure S7). These findings suggest the coexistence of PAC as a local computation and IFC as a global computation in the brain, that work cooperatively (Canolty & Knight, 2010; Dimitriadis, 2018). Indeed, previous reports indicate that the brain network among the TP, the CM, and the posterior parietal regions plays a significant role in audiovisual integration in adults (Ohki et al., 2016). However, the current results suggest that these functional computations still remain underdeveloped in adolescence. Taken together, the neurophysiological properties of both the local (the coupling phase) and global computations (IFC) in adolescents may be the key to understanding the functional maturation in adolescence. More precise elucidation of these issues should be investigated in future studies.

### 4.2 The neuronal foundation of delta‐beta coupling and its unique functionality

Several studies have shown that delta‐beta coupling may be a necessary complement to theta‐gamma coupling for the brain (Cannon et al., 2014; Kopell, Ermentrout, Whittington, & Traub, 2000). To date, delta‐beta PAC has been reported in association cortexes, such as the sensorimotor cortex and TP (Arnal, Doelling, & Poeppel, 2015; Ohki et al., 2016). In the association cortexes, delta‐beta coupling as a complement to theta‐gamma coupling maintains patterns of neuronal activity that code for the features of a multisensory object (Dean, Hagan, & Pesaran, 2012; Kramer et al., 2008; Roopun et al., 2008; von Stein, Rappelsberger, Sarnthein, & Petsche, 1999). We believe that the unique functional traits of delta‐beta coupling, which has a special computational advantage, lie in the layered structures of the association cortexes (Cannon et al., 2014; Ohki & Takei, 2018). Thus, delta‐beta coupling is not merely a slower version of theta‐gamma coupling; it has a distinct dynamical structure that is not expressed during gamma activity (Kopell et al., 2000; Kopell, Whittington, & Kramer, 2011).

The function of delta‐beta PAC is hypothesized to be attained from the combination of several key components. The first key elements are intrinsically bursting (IB) excitatory cells in cortical layer 5 because these cells can control cortical state dynamics (Lorincz et al., 2015; Roopun et al., 2006, 2010). Specifically, a small subset of IB cells in layer 5 fire at a slower rhythm, which includes the delta oscillation frequency, and propels the regular initiation of cortical rhythms, such as the “UP” state (Lorincz et al., 2015). Lorincz et al. (2015) found that only IB cells in layer 5 coherently fired at the point of inception of oscillatory activity in local field potentials. Therefore, Lorincz and colleagues described that the IB cells were “the network driver” cells. The IB cells consecutively entrain regular spiking pyramidal cells in layer 2/3 with the aid of fast‐spiking interneurons, such as PV and SOM interneurons, in the same layer. Recent studies have demonstrated that the interactions between three neuronal groups (pyramidal cells, PV neurons, and SOM interneurons) in layer 2/3 produce beta oscillations in vivo (Chen et al., 2017; Veit et al., 2017), and among the three neuron types, induced beta oscillations are most highly correlated with the spiking of SOM cells. Thus, IB cells and the subsequent downstream activation of synaptic currents in layer 2/3 neurons, to which they project, appear to have a significant role in the creation of delta‐beta coupling (Ohki & Takei, 2018).

### 4.3 Developmental neurophysiological changes in the association cortexes

The statistical properties of the delta‐beta coupling phase, such as the mode and circular variance, showed a distinct trend in adolescents. This trend may be related to the developmental change of GABAergic interneurons, which contain GABA_A_ receptor subunits. Based on the evidence obtained from the association cortexes (e.g., the prefrontal cortex) in rodents, primates, and humans, many studies have demonstrated that GABAergic interneurons in the association cortexes show a dramatic change during adolescence (Caballero & Tseng, 2016; Fung et al., 2010; Hashimoto et al., 2010). It offers a possible explanation for the unique trend of the coupling phase in adolescence.

Several studies examining the distribution of GABA_A_ receptor subunits in the prefrontal cortex have revealed distinct developmental expression patterns at the messenger ribonucleic acid (mRNA) and protein levels, and have also confirmed that there is a developmental increase in α1 subunit expression concurrent with a progressive reduction in the α2 and α5 subunits in a layer‐specific manner (Datta et al., 2015; Hashimoto et al., 2010). These findings suggest that a shift from α2‐GABA_A_ and α5‐GABA_A_ receptors to α1‐containing GABA_A_ receptors occurs in the late‐maturing association cortexes during adolescence. Given that α1 subunits confer specific properties to GABA_A_ function (i.e., faster decay times) that ultimately promote fast synaptic inhibition (Bosman, Rosahl, & Brussaard, 2002; Vicini et al., 2001), the developmental shift in the GABA_A_ subunits may have a profound biological impact. However, neurosteroids also have a significant role in brain development, such as the formation of the cytoskeleton (Belelli et al., 2018), and represent another possible cause of unstable GABA_A_ function. In fact, previous studies have reported that pyramidal neurons and GABA_A_ inhibitory neurons in the cortical layer 2/3 are profoundly influenced by endogenous neurosteroids and result in slow to fast phasic synaptic events (Brown et al., 2015, 2016). We hypothesize that these factors may be coupled and create a distinct coupling property.

In our study, we focused on male adolescents who were 16–17 years old, because a previous study demonstrated that marked developmental changes in PAC were often observed in this age range. However, it is not clear if our findings can be extrapolated to female adolescents or a younger age group. Therefore, sex‐ and age‐related effects on the timing of PAC should be elucidated in future studies.

In conclusion, our findings suggest that the timing of PAC is essential for binding audiovisual information and underlies the developmental process in adolescents.

## CONFLICT OF INTEREST

The authors have no conflict of interest to declare.

## AUTHOR CONTRIBUTIONS

T.O. and A.G designed the study. T.O. and A.G. conducted the research with help from R.S., Y.K., M.I., and T.K. Further, T.O. and T.M. analyzed the data. T.O. and T.M. wrote the manuscript with help from A.G., Y.T., R.S., Y.K., M.I., T.H., K.U., M.F., and K.H. Finally, T.O. and T.M assembled and integrated the contributions for the final version.

## Supporting information

Fig S1

Fig S2

Fig S3a‐h

Fig S3i‐p

Fig S3q‐t

Fig S4a‐h

Fig S4i‐p

Fig S4q‐t

Fig S5

Fig S6

Fig S7

Fig S8

Appendix S1

## Data Availability

The data and codes that support the findings of this study are available by contacting the corresponding author (T.O.).
