# Supplementary figures and images for: Timing of phase‐amplitude coupling is essential for neuronal and functional maturation of audiovisual integration in adolescents
Source: Brain Behav. 2020 Apr 27;10(6):e01635. doi: 10.1002/brb3.1635 (PMC7303405; doi:10.1002/brb3.1635)

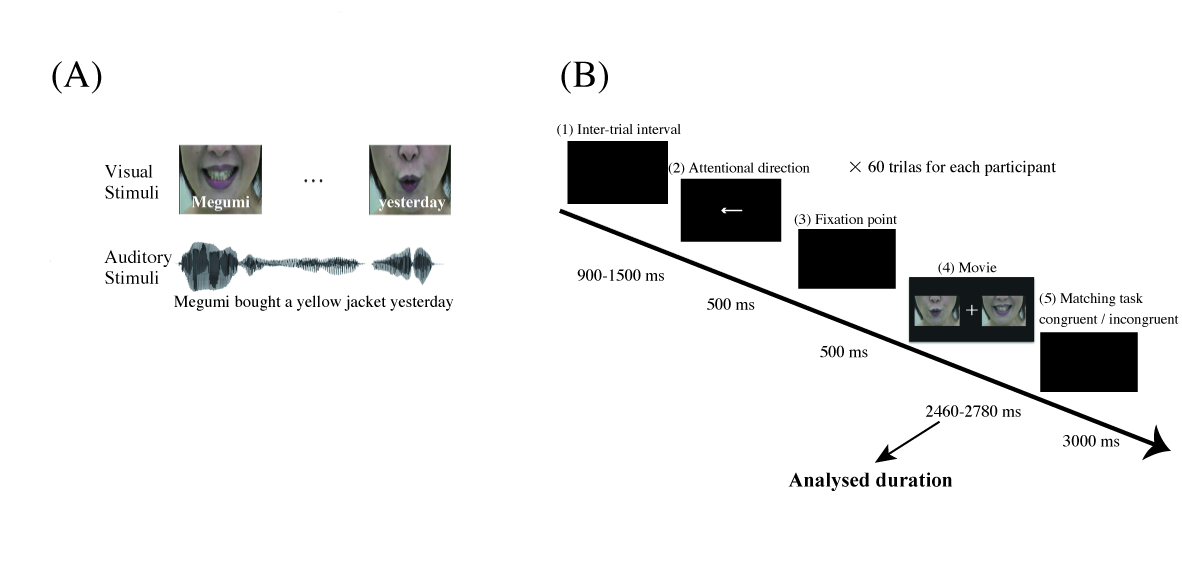

Supplement: Supplementary file 1 — Fig S1 [file BRB3-10-e01635-s001.tif]

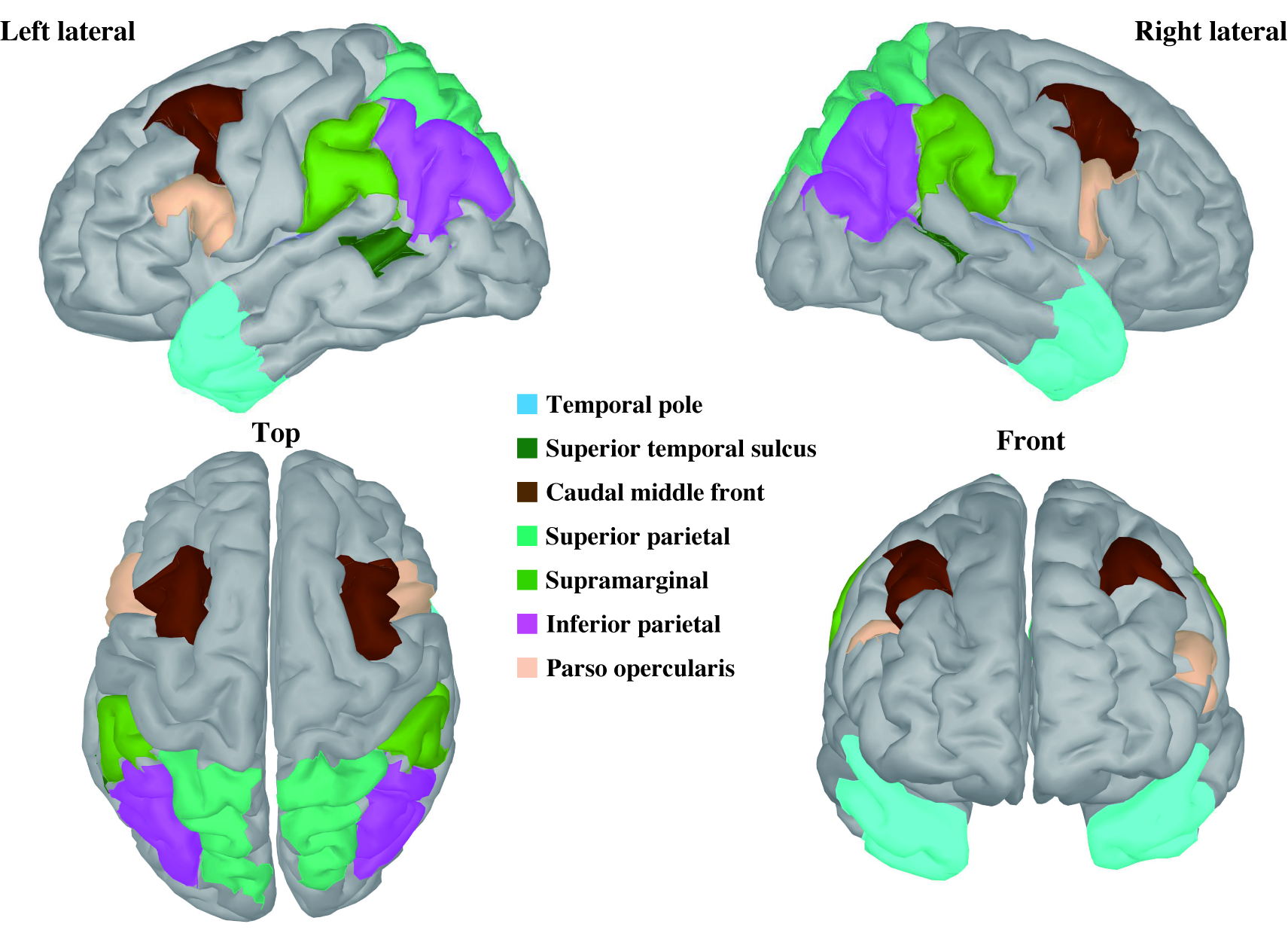

Supplement: Supplementary file 2 — Fig S2 [file BRB3-10-e01635-s002.tif]

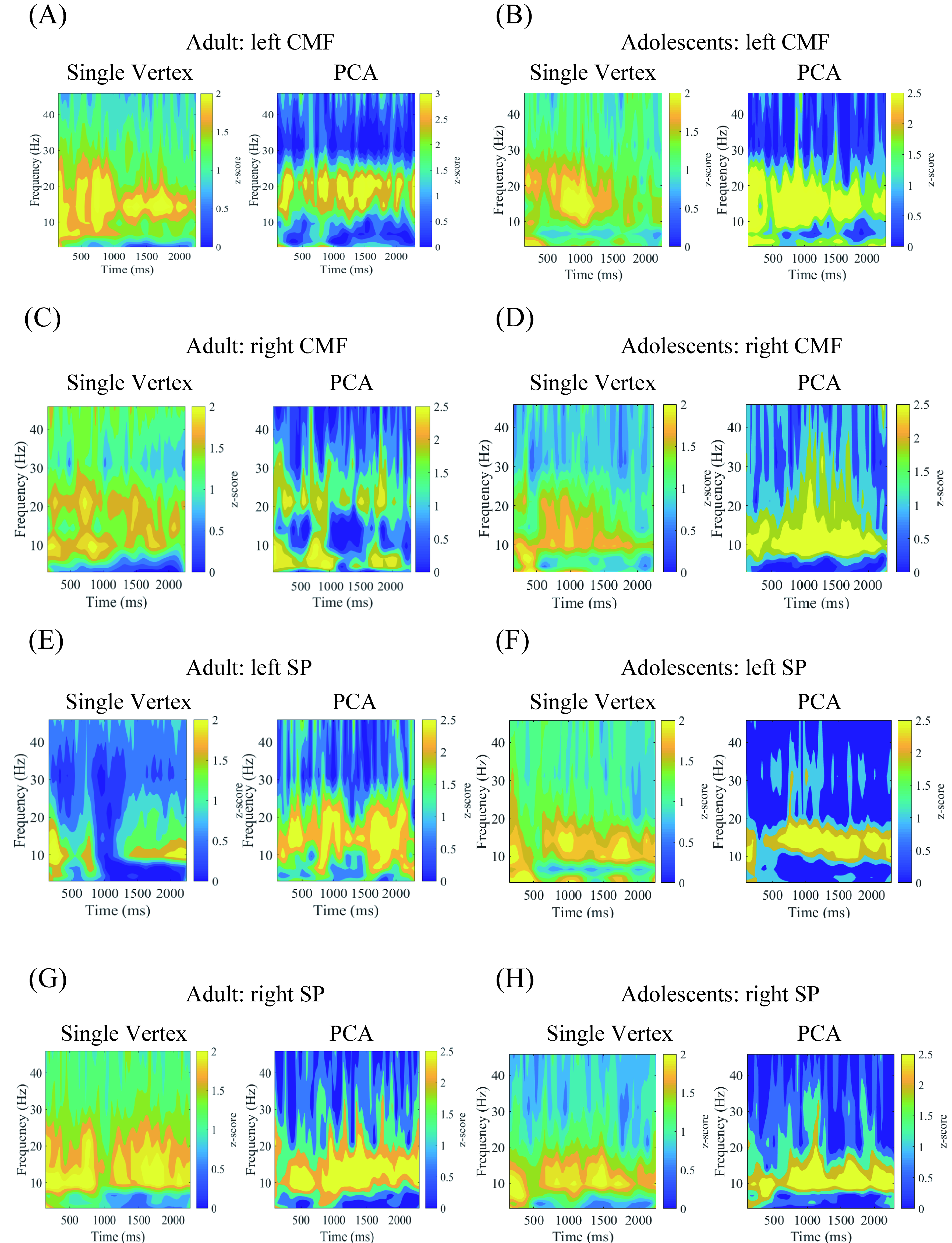

Supplement: Supplementary file 3 — Fig S3a‐h [file BRB3-10-e01635-s003.tif]

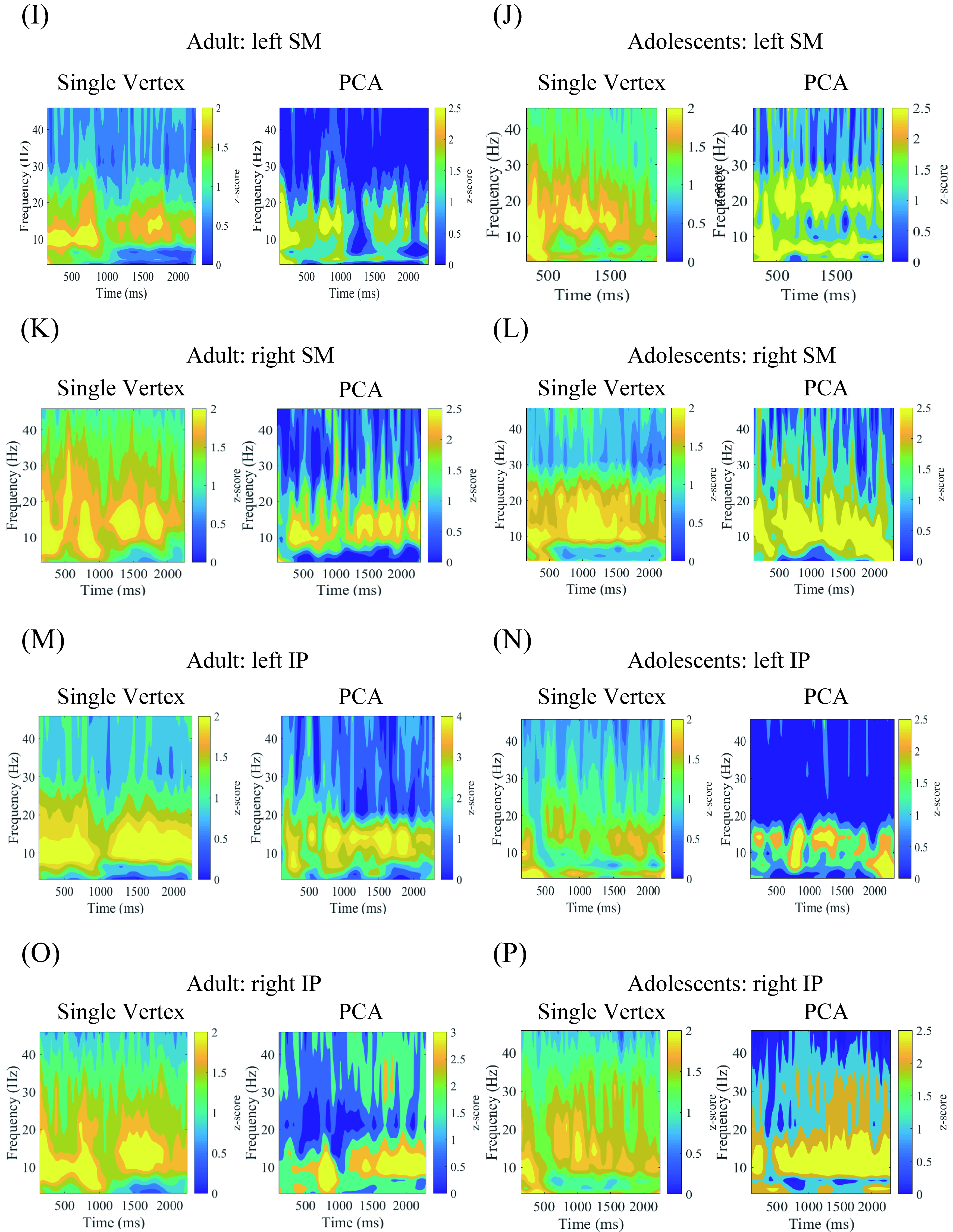

Supplement: Supplementary file 4 — Fig S3i‐p [file BRB3-10-e01635-s004.tif]

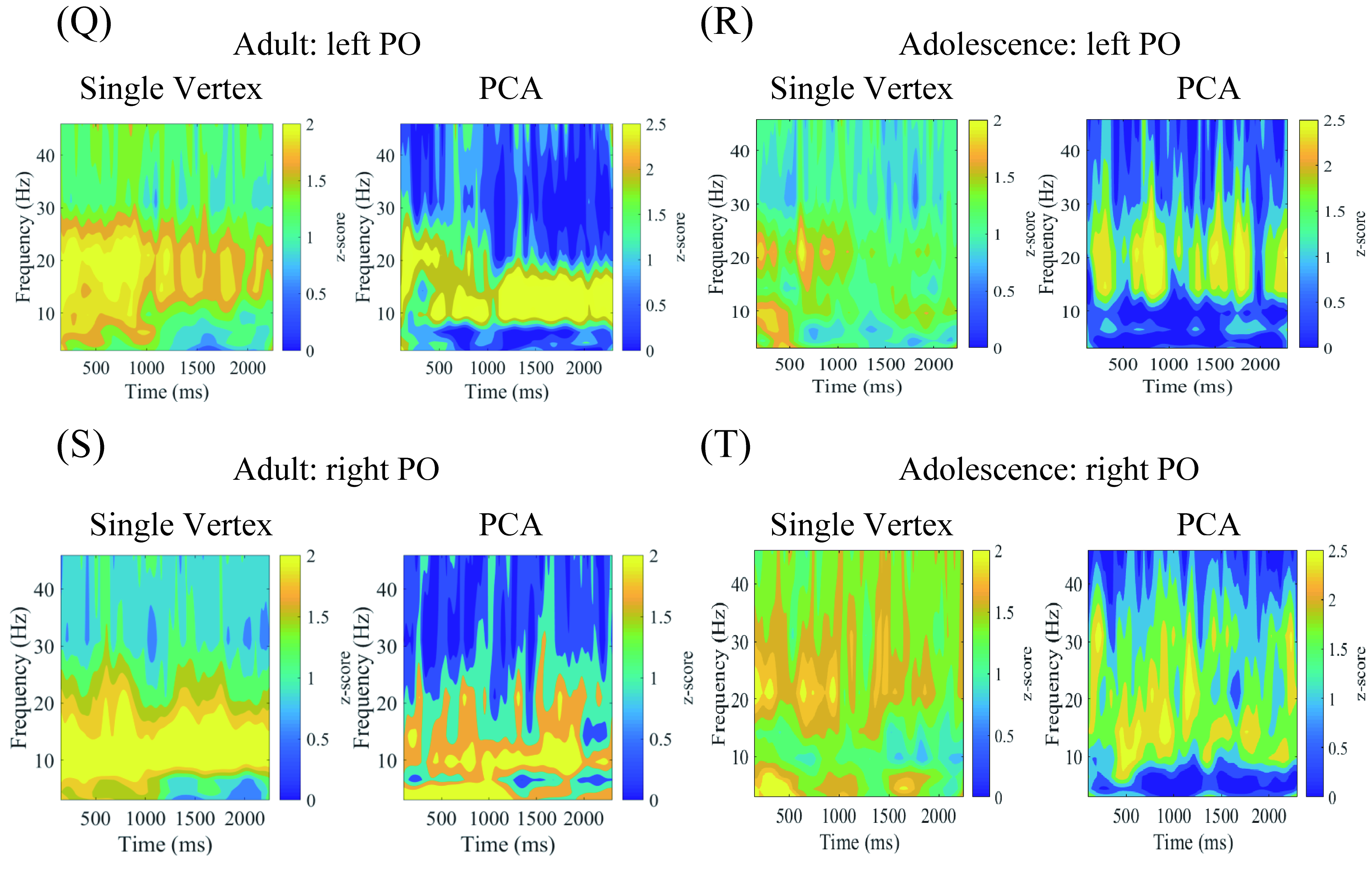

Supplement: Supplementary file 5 — Fig S3q‐t [file BRB3-10-e01635-s005.tif]

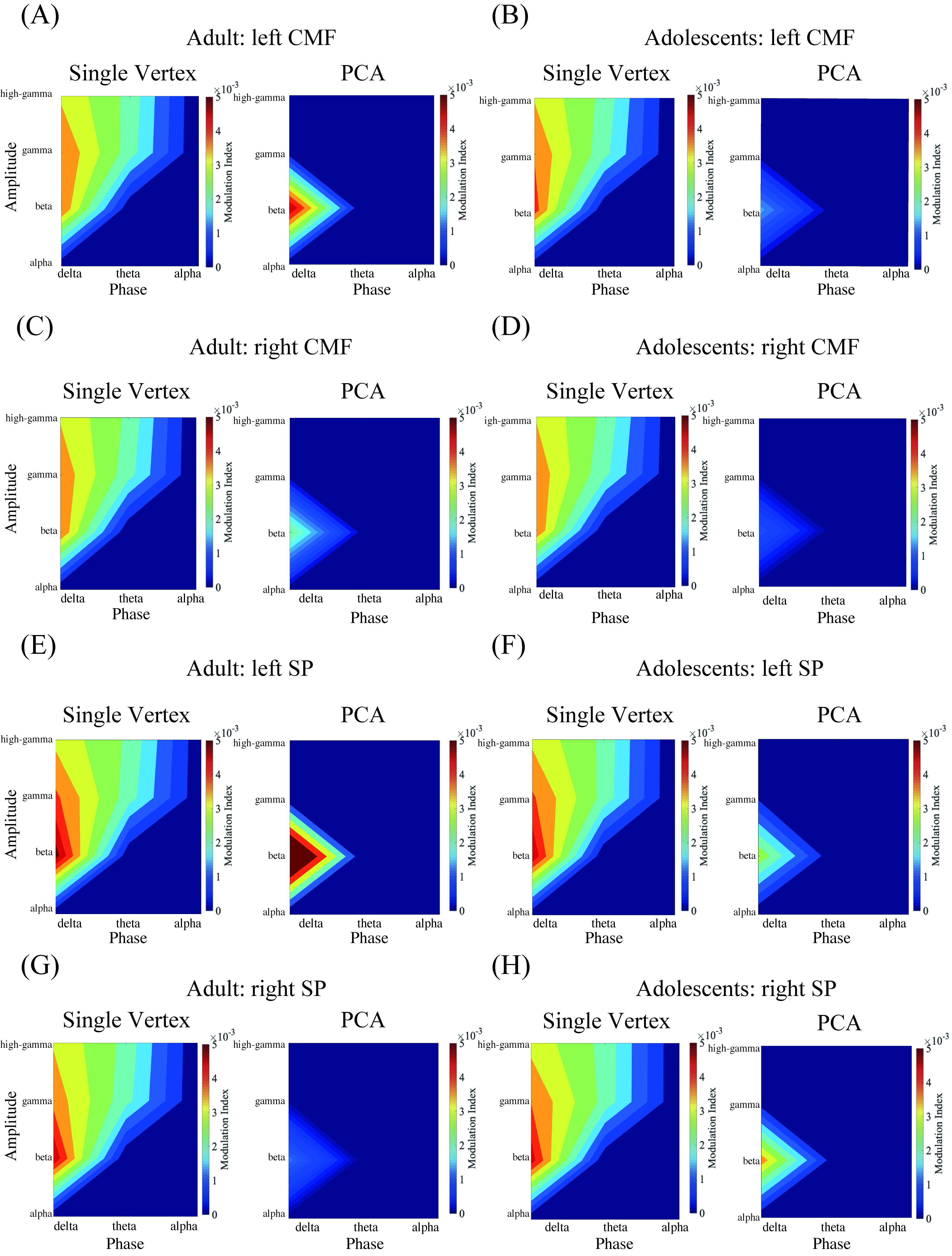

Supplement: Supplementary file 6 — Fig S4a‐h [file BRB3-10-e01635-s006.tif]

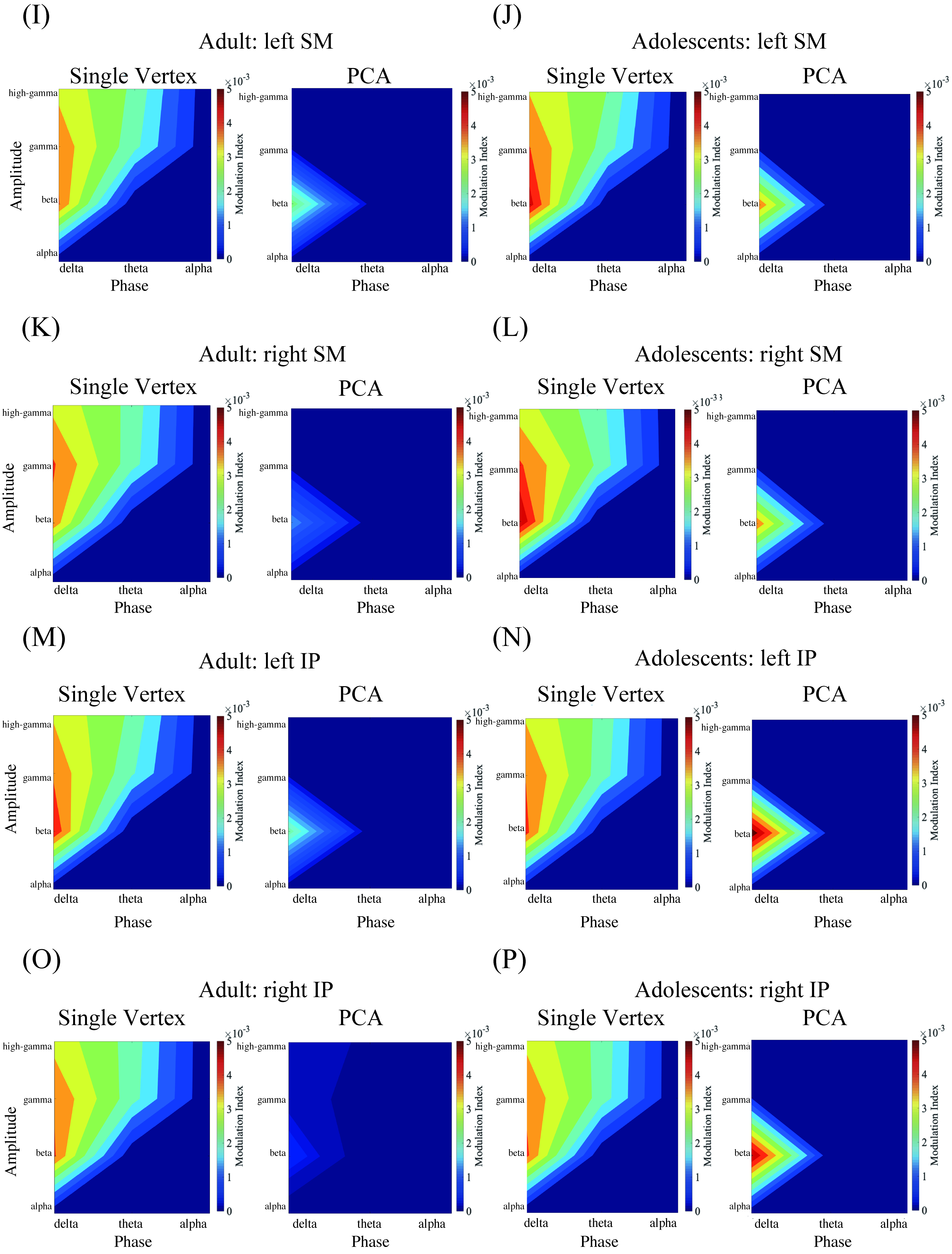

Supplement: Supplementary file 7 — Fig S4i‐p [file BRB3-10-e01635-s007.tif]

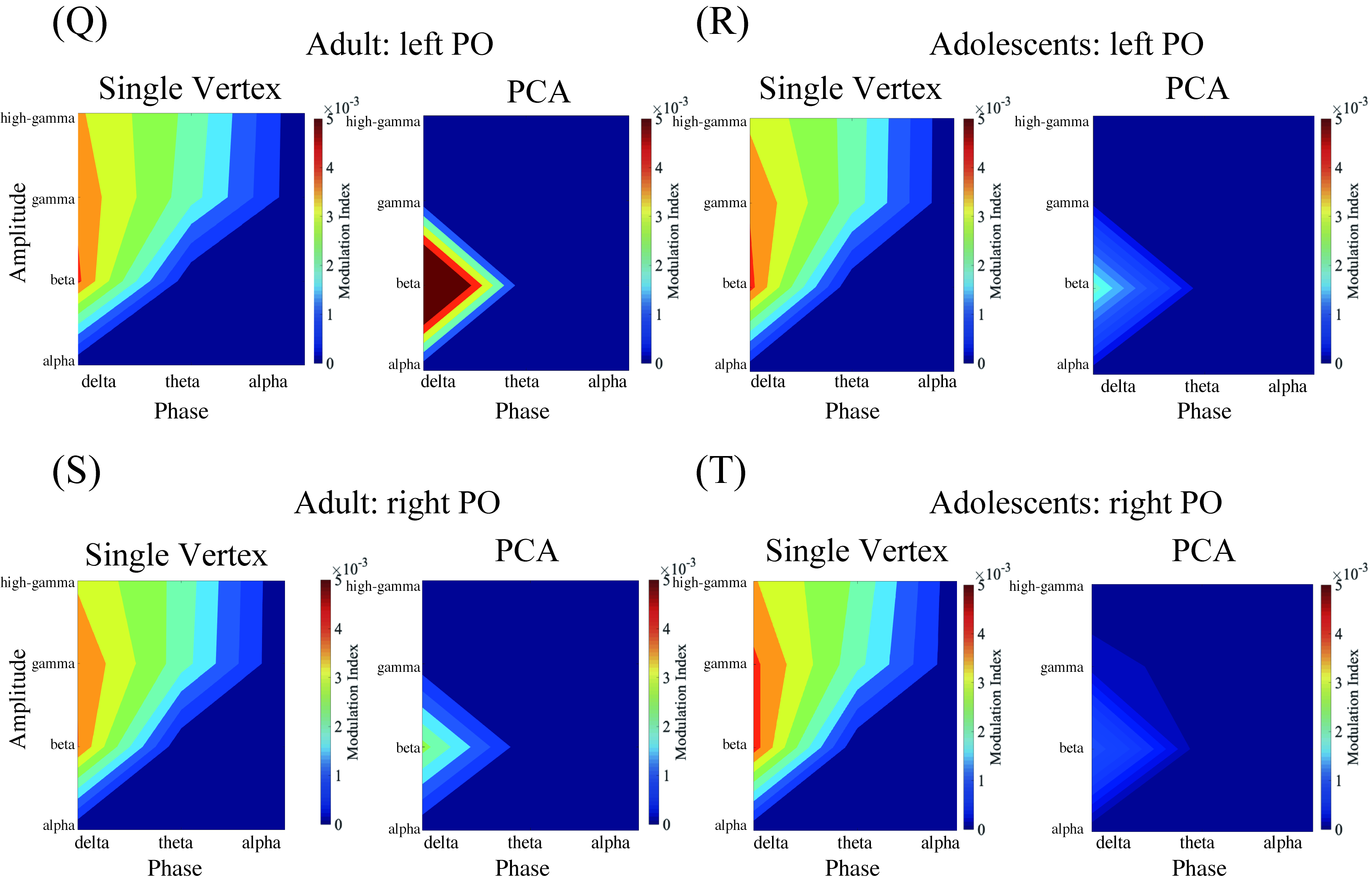

Supplement: Supplementary file 8 — Fig S4q‐t [file BRB3-10-e01635-s008.tif]

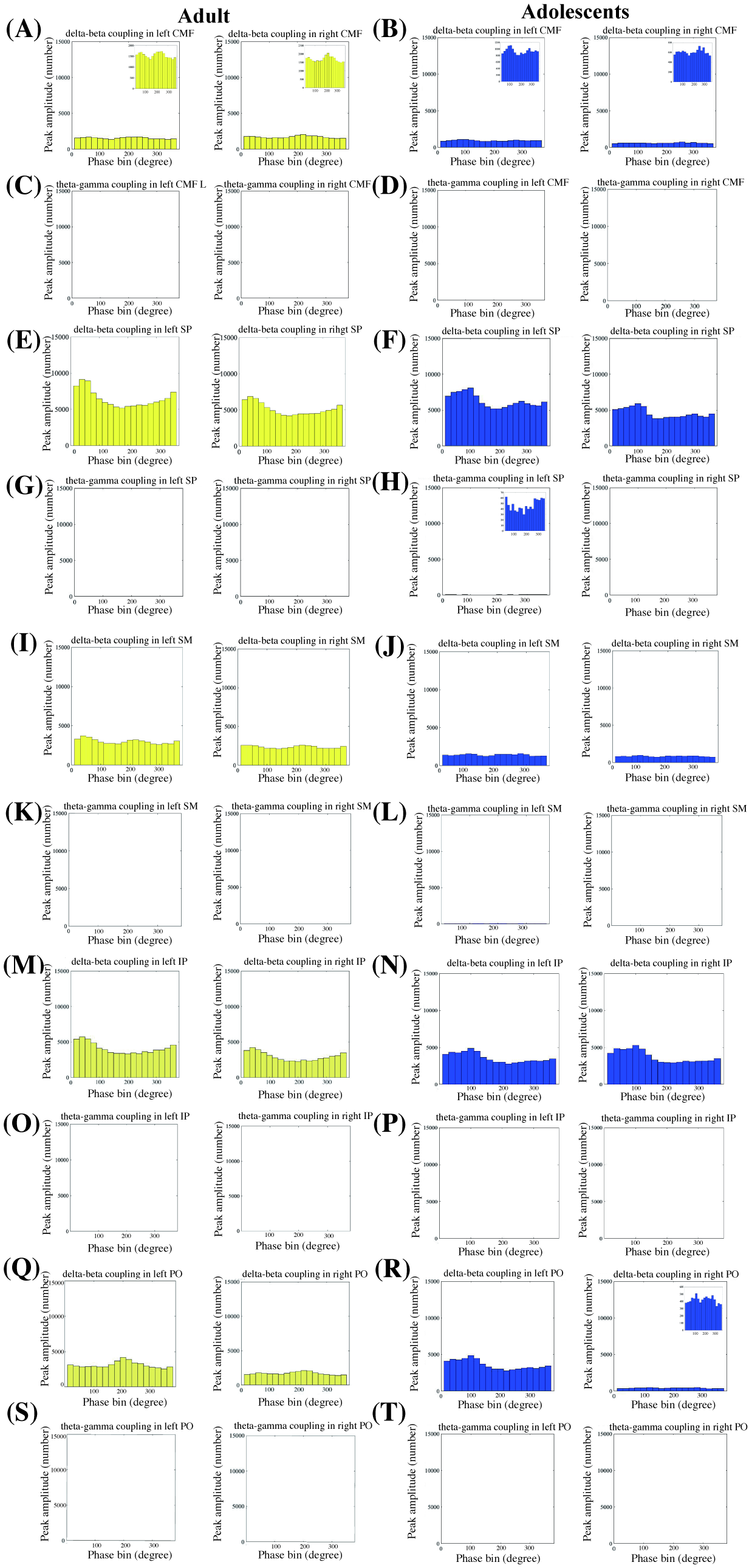

Supplement: Supplementary file 9 — Fig S5 [file BRB3-10-e01635-s009.tif]

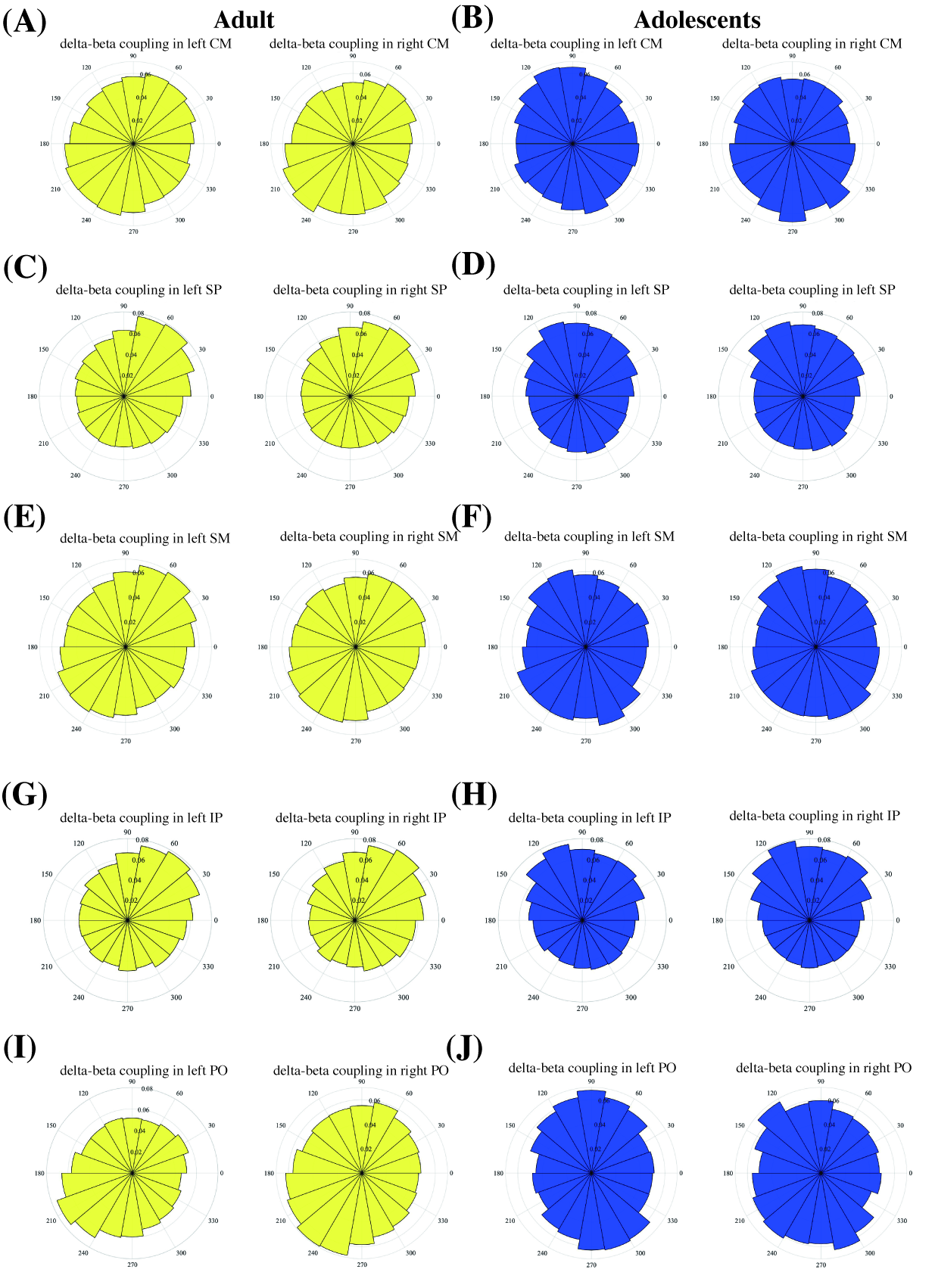

Supplement: Supplementary file 10 — Fig S6 [file BRB3-10-e01635-s010.tif]

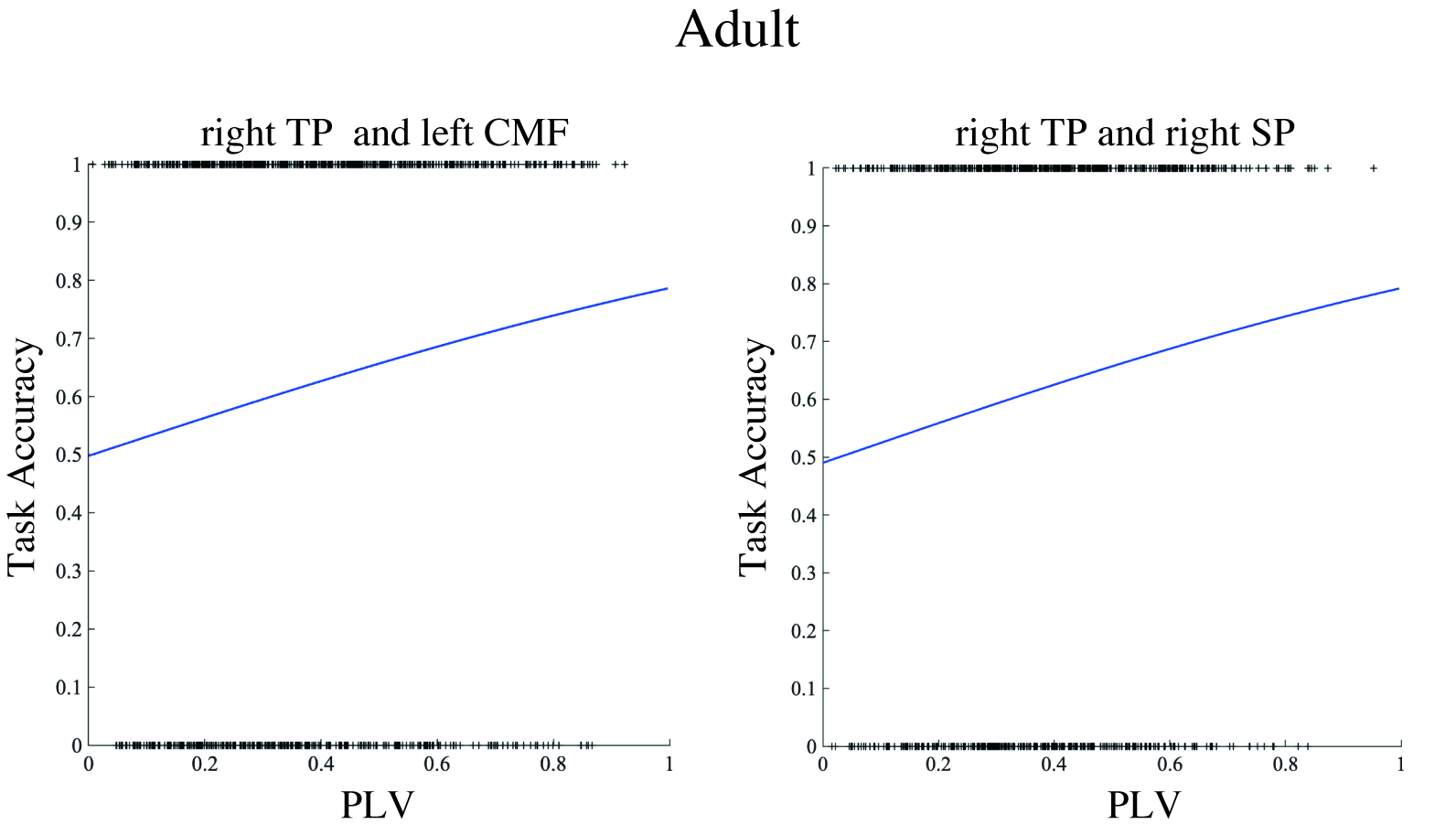

Supplement: Supplementary file 11 — Fig S7 [file BRB3-10-e01635-s011.tif]

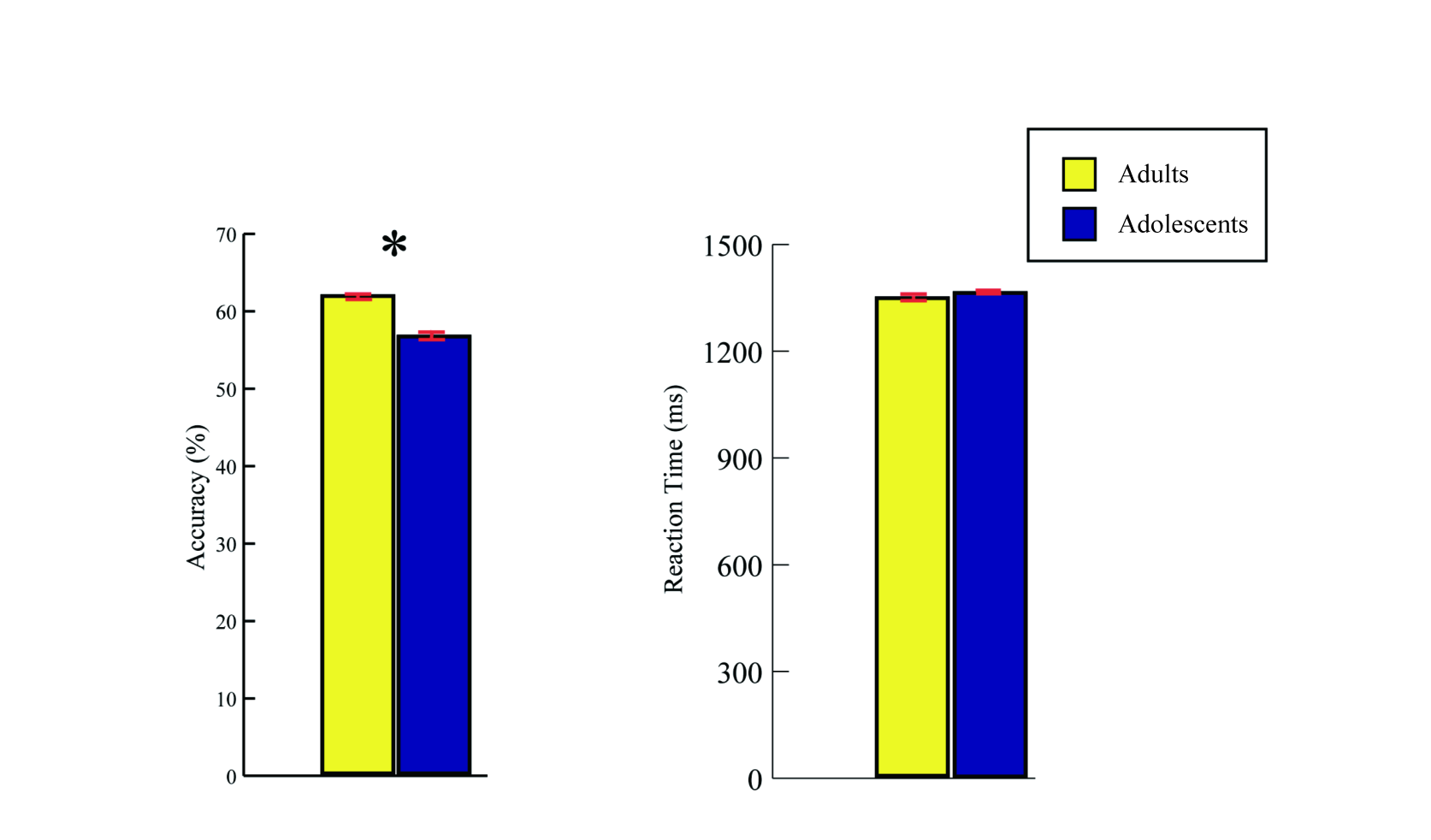

Supplement: Supplementary file 12 — Fig S8 [file BRB3-10-e01635-s012.tif]
