# Supplementary material for: Timing of phase‐amplitude coupling is essential for neuronal and functional maturation of audiovisual integration in adolescents
Source: Brain Behav. 2020 Apr 27;10(6):e01635. doi: 10.1002/brb3.1635 (PMC7303405; doi:10.1002/brb3.1635)
Supplement: Supplementary file 13 — Appendix S1 [file BRB3-10-e01635-s013.docx]

**Timing of phase-amplitude coupling is essential for neuronal and functional maturation of audiovisual integration in adolescents**

Takefumi Ohki, Takeru Matsuda, Atsuko Gunji, Yuichi Takei, Ryusuke Sakuma, Yuu Kaneko, Masumi Inagaki, Takashi Hanakawa, Kazuhiro Ueda, Masato Fukuda, Kazuo Hiraki

**Supporting Materials**

**Supplementary Figure 1**

**Experimental design**. (A) Illustration of the experimental conditions. In each trial, two stimuli are presented simultaneously. The stimuli are presented in a random order across the participants. (B) The experimental procedure: (1) the inter-trial interval (ITI) is 900−1500 ms, (2) the participant views the indicator (← or →), which denotes which movie the participant should focus on; (3) the fixation point is presented for 500 ms; (4) the movie is presented for 2460−2780 ms; and (5) a pair of matching or mismatching words is presented on the screen for 3 seconds. We analysed the signal during the movie section for the source estimation and phase-amplitude coupling. Our movies were 2460 ms to 2780 ms long. Therefore, we analysed the 2400 ms immediately after the start of the movie.

**Supplementary Figure 2**

We focused on seven brain regions: temporal pole (light blue), superior temporal sulcus (dark green), caudal middle front (brown), superior parietal region (emerald green), supramarginal region (light green), inferior parietal region (pink), and parso opercularis (beige). The atlas is based on the *Desikan–Killanany Atlas* (Desikan et al., 2006) and previous studies with a focus on the temporal pole (Ding et al., 2009; Fan et al., 2013; Pascual et al., 2015).

**Supplementary Figure 3**

**Power spectrogram using the complex Morlet wavelet convolution.**

We conducted the convolution for the source estimation data using the complex Morlet wavelet kernel for five regions of interest (ROIs) including the caudal middle frontal (CMF) regions (images A, B, C, and D), superior parietal (SP) (images E, F, G, and H), supramarginal (SM) (images I, J, K, and L), the inferior parietal (IP) regions (images M, N, O, and P), and the parso opercularis (PO) (images Q, R, S, and T). In the left two columns, the results from the adults are depicted. The power spectrograms from the adolescents are shown on the right. In all ROIs, the power of the alpha, beta, and gamma bands were strongly enhanced during our task. Note that we conducted the convolution in two-ways; i.e., one was carried out for the source waveform at a single vertex level and the other was for the principal components (PCA). The single vertex level calculation provided more detailed information for the source space. On the other hand, the calculation using the (PCA) demonstrated more representative spatial information for each ROI. For demonstration purposes, we arbitrarily selected one typical activation pattern from one vertex and one component in each ROI.

**Supplementary Figure 4**

**Comodulograms of the calculated Modulation Index**

These calculations were conducted for the single vertex and for the principal component (PCA) for all the regions of interest (ROIs), including the bilateral caudal middle frontal (CMF) regions (images A, B, C, and D), superior parietal (SP) (images E, F, G, and H), supramarginal (SM) (images I, J, K, and L), the inferior parietal (IP) regions (images M, N, O, and P), and the parso opercularis (PO) (images Q, R, S, and T). In the left and right two columns, the results from the adults and adolescents are shown, respectively. In all ROIs, the most remarkable coupling pattern was the delta-beta coupling. In all ROIs, we confirm that the power of the alpha, beta, and gamma bands were strongly enhanced during our task. Note that there are differences between the calculations at the single vertex and the PCA. Namely, the Comodulograms of the PCA only captured delta-beta coupling. To the contrary, the Modulation Index (MI) at the single vertex base indicated that several coupling patterns did coexist in the source space. These results suggest that the single vertex level calculation provided more detailed information for the source space. However, the calculation using the PCAs demonstrated more representative spatial information for each ROI. For demonstration purposes, we arbitrarily selected one typical activation pattern from one vertex and component in each ROI.

**Supplementary Figure 5**

**Histograms of the coupling phase of delta-beta and theta-gamma coupling.**

The yellow and blue histograms denote the adult and adolescent group, respectively. Consistent with the temporal pole (TP) and superior temporal sulcus (STS), very few trials show theta-gamma coupling. For the sake of brevity, the number of vertices, mode, and circular variance of the delta-beta coupling phase are presented. (A and B) The yellow histograms (adults) and blue histograms (adolescents) represent the number of trials showing delta-beta coupling in the left and right caudal middle frontal (CMF) region. In adults, the mode and circular variance are 240 degrees and 0.038, respectively in the left CMF, and 220 degrees and 0.082, respectively in the right CMF. In adolescents, the mode and circular variance are 100 degrees and 0.032, respectively in the left CMF and 260 degrees and 0.024, respectively in the right CMF. (C and D) The histograms of theta-gamma coupling in the bilateral (i.e., left and right) CMF are depicted. No trial presented theta-gamma coupling in the adult or adolescent groups. (E and F) The number of trials showing delta-beta coupling in the bilateral superior parietal (SP) are depicted. In adults, the mode and circular variance are 40 degrees and 0.031, respectively in the left SP, and 40 degrees and 0.005, respectively in the right SP. However, in adolescents, the mode of the coupling phase in the bilateral SP is 100 degrees, and the circular variance is 0.085 and 0.084 in the left SP and right SP, respectively. (G and H) Theta-gamma coupling in the bilateral SP. No trial demonstrates theta-gamma coupling in adults. In adolescents, only one vertex was detected, for which the mode and circular variance are 20 degrees and 0.103, respectively. (I and J) Histograms of delta-beta coupling in the supramarginal (SM) region. In adults, 60 vertices were detected in the right SM, for which the mode and circular variance are 40 degrees and 0.031, respectively. On the right, 57 vertices were detected. The mode and circular variance are 220 degrees and 0.007, respectively. In adolescents, 30 vertices and 24 vertices in the left SM and right SM, respectively, show delta-beta coupling. In the left SM, the mode is 280 degrees and the circular variance is 0.016. In the right SM, the mode is 100 degrees and the circular variance is 0.009. (K and L) No vertex shows theta-gamma coupling in the bilateral SM in either group. (M and N) The number of trials showing delta-beta coupling in the bilateral inferior parietal (IP) are depicted. In adults, the mode and the circular variance are 40 degrees and 0.119, respectively for the left IP, and 40 degrees and 0.136, respectively for the right IP. However, in adolescents, the mode of the coupling phase in the bilateral IP is 100 degrees, and the circular variance is 0.117 and 0.140 in the left IP and right IP, respectively. (O and P) No vertex shows theta-gamma coupling in the IP of the adults or adolescents. (Q and R) Histograms of delta-beta coupling in the parso opercularis (PO) are depicted. In adults, 62 vertices and 34 vertices in the left and right PO, respectively are detected. The mode and circular variance are 200 degrees and 0.088, respectively in the left PO, and 220 degrees and 0.063, respectively in the right PO. In the adolescents, 20 vertices and 9 vertices were detected in the left PO and right PO, respectively. The mode and circular variance are 100 degrees and 0.117, respectively in the left PO, and 120 degrees and 0.050, respectively in the right PO. (S and T) No vertex shows theta-gamma coupling in the PO region in either group. Note that these figures were depicted in order to show the peak of the coupling phase (the scale of y-axis was modified).

**Supplementary Figure 6**

Rose plots of the delta-beta coupling phase in five brain regions. The yellow and blue plots denote the adult group and adolescent group, respectively. The inner number is the probability (i.e. the number of trials in each bin divided by the total number of trials in each brain region). (A and B) Rose plots of the trials showing delta-beta coupling in the bilateral caudal middle frontal (CMF) region. In the adult group, the coupling phase occurs at 240 degrees in the left CMF and 220 degrees in the right CMF. The blue rose plots present the probability distribution of the coupling phase in adolescents. In adolescents, the mode is 100 degrees and 260 degrees in the left and right CMF, respectively. (C and D) Rose plots of delta-beta coupling in the superior parietal (SP) region. The mode in the bilateral SP is 40 degrees in adults. In adolescents, the mode is 100 degrees in the bilateral SP. (E and F) The rose plots of delta-beta coupling in the supramarginal (SM) region. In adults, the mode is 40 degrees and 220 degrees in the left and right SM region, respectively. In adolescents, the mode is 280 degrees and 100 degrees in the left and right SM region, respectively. (G and H) The number of trials showing delta-beta coupling in the bilateral IP region. In adults, the mode is 40 degrees in the left inferior parietal (IP) and 40 degrees in the right IP. However, in adolescents, the mode of the coupling phase is 100 degrees in the bilateral IP. (I and J) Rose plots of the parso opercularis (PO). The left rose plots present the delta-beta coupling phase in the adults. The mode in the left and right PO is 200 degrees and 220, respectively. By contrast, the mode in the bilateral PO in the adolescents is 100 degrees.

We conducted the likelihood ratio test as the multinomial test for the regions of interest (ROIs) in each of the five brain regions. The findings revealed that the statistical properties of the delta-beta coupling phase in each ROI between the two groups were significantly different (p < 10^-6^). Based on the statistical properties of the delta-beta coupling phase, we determined the general trend of our data. We detected more vertices with a direction at a specific delta phase in the adults than in the adolescents. For instance, the mode of the coupling phase tended to be delayed by approximately 30–40 ms in most brain regions. These results suggest that the coupling phase has certain specific directionality as the brain matures.

**Supplementary Figure 7**

**Logistic regression analysis with phase-locking values.**

We conducted a logistic regression analysis; i.e., the predictor variable was the phase-locking value (PLV) and the objective variable was the success or failure of each trial (1 or 0). To calculate the PLV, we chose the right temporal pole (TP) as the seed region, based on the results of a logistic regression analysis with the coupling phase. As a result, we found PLV of the theta oscillation was only a significant predictor in adults (p < 0.05 with Bonferroni correction (p-values multiplied by 13*5 [the number of the combinations of the regions and the number of the principal components [PCs)]). These results suggest that when PLV in theta oscillations between the right TP, the left caudal middle frontal (CMF) region, and the right superior parietal (SP) became higher, the higher the task score became. The blue line in the figure is an estimated regression line.

**Behavioural analysis**

Using the generalised linear mixed model, we analysed the task accuracy. We used a binomial distribution for the accuracy data. For the reaction time (RT) data, we first tested whether our RT data fitted an exponentially modified Gaussian (i.e. ex-Gaussian) distribution. We demonstrated that the RT data for both groups followed an identical ex-Gauss distribution. The likelihood ratio test was subsequently applied to confirm whether the RT data between the two groups was significantly different. We found a statistically significant difference in task accuracy between the adult and adolescent groups, but no statistically significant difference in the RT.

**Supplementary Figure 8**

**Behavioural results**. The correct responses and reaction times are averaged across each group. The yellow bar denotes the adult group and the blue bar denotes the adolescent group. A significant difference in the accuracy indicates that adolescents were less successful in processing audiovisual information than the adults. The left bar in the left figure indicates the 95% confidence interval for the binomial distribution. The right bar in the right figure denotes the standard error.

* p < 0.05.

**Supplementary Table 1** Demographic characteristics of the participants

|  | Adults | Adolescents |
| --- | --- | --- |
| Chronological age, y, mean (range) | 25.6 (22–35) | 16.8 (16–17) |
| WAIS-Ⅲ | VIQ = 122.3 (8.20)  PIQ = 115.7 (7.45) | VIQ = 121.2 (6.4)  PIQ = 112.3 (7.9) |
| SCQ | — | 2.16 (2.03) |
| AQ | 17.53 (6.38) | — |
| ADHD-rs | Inattention = 6.3 (5.92)  Hyperactivity = 4.13 (4.31) | Inattention = 3.0 (3.74)  Hyperactivity = 0.92 (2.21) |

*Note*: The numbers in the parentheses indicate the standard deviation, unless otherwise indicated.

ADHD-rs: Attention Deficit-Hyperactivity Disorder Rating Scale, AQ: Autism Questionnaire, PIQ: Performance Intelligence Quotient, SCQ: Social communication questionnaire, VIQ: Verbal Intelligence Quotient, WAIS-III: Wechsler Adult Intelligence Scale

**Supplementary Table 2.** **Percentage of trials with a significant Modulation Index in each region of interest**

| Region  Group name  (no. of vertices) | Coupling pattern | Percentage of trials with significant MI (%) |
| --- | --- | --- |
| CMF L  Adults  (196) | Delta-beta | 99.04 |
|  | Theta-gamma | 88.88 |
|  | Delta-gamma | 99.35 |
|  | Delta-high gamma | 99.95 |
|  | Theta-high gamma | 98.77 |
| CMF L  Adolescents  (196) | Delta-beta | 99.20 |
|  | Theta-gamma | 89.28 |
|  | Delta-gamma | 99.39 |
|  | Delta-high gamma | 99.96 |
|  | Theta-high gamma | 98.89 |
| CMF R  Adults  (214) | Delta-beta | 99.05 |
|  | Theta-gamma | 88.99 |
|  | Delta-gamma | 99.28 |
|  | Delta-high gamma | 99.94 |
|  | Theta-high gamma | 98.73 |
| CMF R  Adolescents  (214) | Delta-beta | 99.07 |
|  | Theta-gamma | 88.57 |
|  | Delta-gamma | 99.37 |
|  | Delta-high gamma | 99.96 |
|  | Theta-high gamma | 98.74 |
| SP L  Adults  (439) | Delta-beta | 98.96 |
|  | Theta-gamma | 88.56 |
|  | Delta-gamma | 99.35 |
|  | Delta-high gamma | 99.95 |
|  | Theta-high gamma | 98.72 |
| SP L  Adolescents  (439) | Delta-beta | 99.06 |
|  | Theta-gamma | 88.95 |
|  | Delta-gamma | 99.36 |
|  | Delta-high gamma | 99.95 |
|  | Theta-high gamma | 98.72 |
| SP R  Adults  (435) | Delta-beta | 98.96 |
|  | Theta-gamma | 88.60 |
|  | Delta-gamma | 99.31 |
|  | Delta-high gamma | 99.94 |
|  | Theta-high gamma | 98.66 |
| SP R  Adolescents  (435) | Delta-beta | 99.15 |
|  | Theta-gamma | 88.82 |
|  | Delta-gamma | 99.36 |
|  | Delta-high gamma | 99.95 |
|  | Theta-high gamma | 98.70 |
| SM L  Adults  (301) | Delta-beta | 98.86 |
|  | Theta-gamma | 88.79 |
|  | Delta-gamma | 99.32 |
|  | Delta-high gamma | 99.94 |
|  | Theta-high gamma | 98.73 |
| SM L  Adolescence  (301) | Delta-beta | 98.98 |
|  | Theta-gamma | 89.04 |
|  | Delta-gamma | 99.36 |
|  | Delta-high gamma | 99.96 |
|  | Theta-high gamma | 98.70 |
| SM R  Adults  (327) | Delta-beta | 98.96 |
|  | Theta-gamma | 88.62 |
|  | Delta-gamma | 99.33 |
|  | Delta-high gamma | 99.94 |
|  | Theta-high gamma | 98.69 |
| SM R  Adolescents  (327) | Delta-beta | 99.03 |
|  | Theta-gamma | 88.93 |
|  | Delta-gamma | 99.32 |
|  | Delta-high gamma | 99.94 |
|  | Theta-high gamma | 98.70 |
| IP L  Adult  (428) | Delta-beta | 98.96 |
|  | Theta-gamma | 88.46 |
|  | Delta-gamma | 99.32 |
|  | Delta-high gamma | 99.95 |
|  | Theta-high gamma | 98.73 |
| IP L  Adolescents  (428) | Delta-beta | 99.08 |
|  | Theta-gamma | 88.96 |
|  | Delta-gamma | 99.35 |
|  | Delta-high gamma | 99.96 |
|  | Theta-high gamma | 98.78 |
| IP R  Adults  (456) | Delta-beta | 98.98 |
|  | Theta-gamma | 88.57 |
|  | Delta-gamma | 99.33 |
|  | Delta-high gamma | 99.95 |
|  | Theta-high gamma | 98.62 |
| IP R  Adolescents  (456) | Delta-beta | 99.15 |
|  | Theta-gamma | 88.76 |
|  | Delta-gamma | 99.37 |
|  | Delta-high gamma | 99.95 |
|  | Theta-high gamma | 98.76 |
| PO L  Adults  (153) | Delta-beta | 99.12 |
|  | Theta-gamma | 89.06 |
|  | Delta-gamma | 99.43 |
|  | Delta-high gamma | 99.95 |
|  | Theta-high gamma | 98.78 |
| PO L  Adolescents  (153) | Delta-beta | 99.13 |
|  | Theta-gamma | 89.11 |
|  | Delta-gamma | 99.33 |
|  | Delta-high gamma | 98.88 |
|  | Theta-high gamma | 99.95 |
| PO R  Adults  (136) | Delta-beta | 99.09 |
|  | Theta-gamma | 88.71 |
|  | Delta-gamma | 99.29 |
|  | Delta-high gamma | 99.95 |
|  | Theta-high gamma | 98.71 |
| PO R  Adolescents  (136) | Delta-beta | 99.18 |
|  | Theta-gamma | 88.93 |
|  | Delta-gamma | 99.39 |
|  | Delta-high gamma | 99.96 |
|  | Theta-high gamma | 98.74 |
| TP L  Adults  (278) | Delta-gamma | 99.28 |
|  | Delta-high gamma | 99.94 |
|  | Theta-high gamma | 98.75 |
| TP L  Adolescents  (278) | Delta-gamma | 99.36 |
|  | Delta-high gamma | 99.95 |
|  | Theta-high gamma | 98.77 |
| TP R  Adults  (316) | Delta-gamma | 99.34 |
|  | Delta-high gamma | 99.95 |
|  | Theta-high gamma | 98.77 |
| TP R  Adolescents  (316) | Delta-gamma | 99.35 |
|  | Delta-high gamma | 99.95 |
|  | Theta-high gamma | 98.79 |
| STS L  Adults  (96) | Delta-gamma | 99.38 |
|  | Delta-high gamma | 99.95 |
|  | Theta-high gamma | 98.66 |
| STS L  Adolescents  (96) | Delta-gamma | 99.33 |
|  | Delta-high gamma | 99.95 |
|  | Theta-high gamma | 98.79 |
| STS R  Adults  (73) | Delta-gamma | 99.31 |
|  | Delta-high gamma | 99.95 |
|  | Theta-high gamma | 98.69 |
| STS R  Adolescents  (73) | Delta-gamma | 99.34 |
|  | Delta-high gamma | 99.96 |
|  | Theta-high gamma | 98.68 |

CMF: caudal middle frontal, IP: inferior parietal, L: left, PO: parso opercularis, R: right, SM: supramarginal, SP: superior parietal, STS: superior temporal sulcus

**Supplementary Table 3.** Statistical properties of the coupling phase

| Region  Group name  (no. of vertices) | Coupling pattern | Number of vertices | Mode (degree) | Circular variance |
| --- | --- | --- | --- | --- |
| CMF L  Adults  (196) | Delta-beta | 31 | 240 | 0.038 |
|  | Theta-gamma | 0 | — | — |
|  | Delta-gamma | 0 | — | — |
|  | Delta-high gamma | 0 | — | — |
|  | Theta-high gamma | 0 | — | — |
| CMF L Adolescents  (196) | Delta-beta | 20 | 100 | 0.032 |
|  | Theta-gamma | 0 | — | — |
|  | Delta-gamma | 0 | — | — |
|  | Delta-high gamma | 0 | — | — |
|  | Theta-high gamma | 0 | — | — |
| CMF R  Adults  (214) | Delta-beta | 34 | 220 | 0.082 |
|  | Theta-gamma | 0 | — | — |
|  | Delta-gamma | 0 | — | — |
|  | Delta-high gamma | 0 | — | — |
|  | Theta-high gamma | 0 | — | — |
| CMF R  Adolescents  (196) | Delta-beta | 13 | 260 | 0.024 |
|  | Theta-gamma | 0 | — | — |
|  | Delta-gamma | 0 | — | — |
|  | Delta-high gamma | 0 | — | — |
|  | Theta-high gamma | 0 | — | — |
|  | Delta-beta | 129 | 40 | 0.031 |
| SP L  Adults  (439) | Theta-gamma | 0 | — | — |
|  | Delta-gamma | 0 | — | — |
|  | Delta-high gamma | 0 | — | — |
|  | Theta-high gamma | 0 | — | — |
| SP L  Adolescents  (439) | Delta-beta | 135 | 100 | 0.085 |
|  | Theta-gamma | 1 | 20 | 0.103 |
|  | Delta-gamma | 3 | 300 | 0.071 |
|  | Delta-high gamma | 0 | — | — |
|  | Theta-high gamma | 0 | — | — |
| SP R  Adults  (435) | Delta-beta | 102 | 40 | 0.005 |
|  | Theta-gamma | 0 | — | — |
|  | Delta-gamma | 1 | 360 | 0.111 |
|  | Delta-high gamma | 0 | — | — |
|  | Theta-high gamma | 0 | — | — |
| SP R  Adolescents  (435) | Delta-beta | 98 | 100 | 0.084 |
|  | Theta-gamma | 0 | — | — |
|  | Delta-gamma | 0 | — | — |
|  | Delta-high gamma | 0 | — | — |
|  | Theta-high gamma | 0 | — | — |
| SM L  Adults  (301) | Delta-beta | 60 | 40 | 0.031 |
|  | Theta-gamma | 0 | — | — |
|  | Delta-gamma | 1 | 280 | 0.107 |
|  | Delta-high gamma | 0 | — | — |
|  | Theta-high gamma | 0 | — | — |
| SM L  Adolescents  (301) | Delta-beta | 30 | 280 | 0.016 |
|  | Theta-gamma | 0 | — | — |
|  | Delta-gamma | 0 | — | — |
|  | Delta-high gamma | 0 | — | — |
|  | Theta-high gamma | 1 | 120 | 0.071 |
| SM R  Adults  (327) | Delta-gamma | 57 | 220 | 0.007 |
|  | Theta-gamma | 0 | — | — |
|  | Delta-gamma | 1 | 40 | 0.105 |
|  | Delta-high gamma | 1 | 100 | 0.102 |
|  | Theta-high gamma | 0 | — | — |
| SM R  Adolescents  (327) | Delta-beta | 24 | 100 | 0.009 |
|  | Theta-gamma | 0 | — | — |
|  | Delta-gamma | 1 | 100 | 0.094 |
|  | Delta-high gamma | 0 | — | — |
|  | Theta-high gamma | 0 | — | — |
| IP L  Adults  (428) | Delta-beta | 82 | 40 | 0.119 |
|  | Theta-gamma | 0 | — | — |
|  | Delta-gamma | 0 | — | — |
|  | Delta-high gamma | 0 | — | — |
|  | Theta-high gamma | 0 | — | — |
| IP L  Adolescents  (428) | Delta-beta | 77 | 100 | 0.117 |
|  | Theta-gamma | 0 | — | — |
|  | Delta-gamma | 1 | 40 | 0.080 |
|  | Delta-high gamma | 0 | — | — |
|  | Theta-high gamma | 0 | — | — |
| IP R  Adults  (456) | Delta-beta | 59 | 40 | 0.136 |
|  | Theta-gamma | 0 | — | — |
|  | Delta-gamma | 1 | 80 | 0.035 |
|  | Delta-high gamma | 0 | — | — |
|  | Theta-high gamma | 0 | — | — |
| IP R  Adolescents  (456) | Delta-beta | 80 | 100 | 0.140 |
|  | Theta-gamma | 0 | — | — |
|  | Delta-gamma | 1 | 60 | 0.093 |
|  | Delta-high gamma | 0 | — | — |
|  | Theta-high gamma | 0 | — | — |
| PO L  Adults  (153) | Delta-beta | 62 | 200 | 0.088 |
|  | Theta-gamma | 0 | — | — |
|  | Delta-gamma | 0 | — | — |
|  | Delta-high gamma | 0 | — | — |
|  | Theta-high gamma | 0 | — | — |
| PO L  Adolescents  (153) | Delta-beta | 20 | 100 | 0.117 |
|  | Theta-gamma | 0 | — | — |
|  | Delta-gamma | 0 | — | — |
|  | Delta-high gamma | 0 | — | — |
|  | Theta-high gamma | 0 | — | — |
| PO R  Adults  (136) | Delta-beta | 34 | 220 | 0.063 |
|  | Theta-gamma | 0 | — | — |
|  | Delta-gamma | 1 | 100 | 0.099 |
|  | Delta-high gamma | 0 | — | — |
|  | Theta-high gamma | 0 | — | — |
| PO R  Adolescents  (136) | Delta-beta | 9 | 120 | 0.050 |
|  | Theta-gamma | 0 | — | — |
|  | Delta-gamma | 0 | — | — |
|  | Delta-high gamma | 0 | — | — |
|  | Theta-high gamma | 0 | — | — |
| TP L  Adults  (278) | Delta-gamma | 33 | 220 | 0.070 |
|  | Delta-high gamma | 0 | — | — |
|  | Theta-high gamma | 0 | — | — |
|  | Delta-gamma | 34 | 260 | 0.090 |
|  | Delta-high gamma | 0 | — | — |
| TP L  Adolescents  (278) | Theta-high gamma | 0 | — | — |
|  | Delta-gamma | 0 | — | — |
|  | Delta-high gamma | 0 | — | — |
| TP R  Adults  (316) | Theta-high gamma | 0 | — | — |
|  | Delta-gamma | 0 | — | — |
|  | Delta-high gamma | 0 | — | — |
| TP R  Adolescents  (316) | Theta-high gamma | 0 | — | — |
|  | Delta-gamma | 0 | — | — |
|  | Delta-high gamma | 0 | — | — |
| STS L  Adults  (96) | Theta-high gamma | 0 | — | — |
|  | Delta-gamma | 0 | — | — |
|  | Delta-high gamma | 0 | — | — |
| STS L Adolescents  (96) | Theta-high gamma | 0 | — | — |
|  | Delta-gamma | 0 | — | — |
|  | Delta-high gamma | 0 | — | — |
| STS R  Adults  (73) | Theta-high gamma | 0 | — | — |
|  | Delta-gamma | 0 | — | — |
|  | Delta-high gamma | 0 | — | — |
| STS R Adolescents  (73) | Theta-high gamma | 0 | — | — |
|  | Delta-gamma | 0 | — | — |
|  | Delta-high gamma | 0 | — | — |

CMF: caudal middle frontal region, IP: inferior parietal region, L: left, PO: parso opercularis, R: right, SM: supramarginal region, SP: superior parietal region, STS: superior temporal sulcus, TP: temporal pole

**References**

1. Desikan, R. S., Segonne, F., Fischl, B., Quinn, B. T., Dickerson, B. C., Blacker, D., Bucker, R. L., Dale, A. M., Maguire, R. P., Hyman, B. T., Albert, M. S., Killiany R. J. 2006. An automated labelling system for subdividing the human cerebral cortex on MRI scans into gyral based regions of interest. *Neuroimage*, 31, 968–980.
2. Ding, S. L., van Hoesen, G. W., Cassell, M. D., Poremba, A. 2009. Parcellation of human temporal polar cortex: a combined analysis of multiple cytoarchitectonic, chemoarchitectonic, and pathological markers. *The Journal of Comparative Neurology*, 514, 595–623. http://dx.doi.org/10.1002/cne.22053
3. Fan, L., Wang, J., Zhang, Y., Han, W., Yu, C., Jiang, T. 2014. Connectivity-based parcellation of the human temporal pole using diffusion tensor imaging. *Cerebral Cortex*, 24, 3365–3378. http://dx.doi.org/10.1093/cercor/bht196
4. Pascual, B., Masdeu, J. C., Hollenbeck, M., Makris, N., Insausti, R., Ding, S. L., Dickerson, B. C. 2015. Large-scale brain networks of the human left temporal pole: a functional connectivity MRI study. *Cerebral Cortex*, 25, 680–702. http://dx.doi.org/10.1093/cercor/bht260
